# Error-Related Potentials in Reinforcement Learning-Based Brain-Machine Interfaces

**DOI:** 10.3389/fnhum.2022.806517

**Published:** 2022-06-24

**Authors:** Aline Xavier Fidêncio, Christian Klaes, Ioannis Iossifidis

**Affiliations:** ^1^Robotics and BCI Laboratory, Institute of Computer Science, Ruhr West University of Applied Sciences, Mülheim an der Ruhr, Germany; ^2^KlaesLab, Department of Neurosurgery, University Hospital Knappschaftskrankenhaus Bochum GmbH, Bochum, Germany; ^3^Faculty of Electrical Engineering and Information Technology, Ruhr-University Bochum, Bochum, Germany

**Keywords:** error-related potentials, reinforcement learning, EEG, brain-machine (computer) interface, electroencephalography, self-organization

## Abstract

The human brain has been an object of extensive investigation in different fields. While several studies have focused on understanding the neural correlates of error processing, advances in brain-machine interface systems using non-invasive techniques further enabled the use of the measured signals in different applications. The possibility of detecting these error-related potentials (ErrPs) under different experimental setups on a single-trial basis has further increased interest in their integration in closed-loop settings to improve system performance, for example, by performing error correction. Fewer works have, however, aimed at reducing future mistakes or learning. We present a review focused on the current literature using non-invasive systems that have combined the ErrPs information specifically in a reinforcement learning framework to go beyond error correction and have used these signals for learning.

## 1. Introduction

Reinforcement learning (RL) problems involve training a so-called agent by allowing it to interact with the environment based on trial-and-error to learn the optimal policy (Sutton and Barto, 2018). For each action taken, the agent receives a numerical reward, and its goal is to maximize its total return. Hence, by collecting knowledge about the environment through interactions, the agent aims at finding the sequence of actions, i.e., policy, for that environment which will lead to maximum accumulated reward in the long term. Unlike the typical supervised and unsupervised learning approaches, RL algorithms do not rely on available labeled or even historical data. Instead, they are based only on evaluative feedback, and this framework has proven suitable for different kinds of applications (Silver, 2015).

A challenge in specifying a reinforcement learning problem involves a meaningful reward function definition. The reward function defines the RL goal and directly impacts learning performance. However, defining a reward function is not a trivial task and might require expert knowledge about both the task and environment. An intuitive alternative to the design of complex reward functions has been proposed in the last years, using a specific neural activity signal generated in the human brain upon erroneous events occurrence. These signals are time-locked to the error-onset event and referred to as error-related potentials (ErrPs). It is possible to measure these signals using non-invasive techniques such as electroencephalography (EEG), and several works have demonstrated that different experimental tasks can elicit ErrPs. Moreover, ErrPs can be reliably detected on a single-trial basis, which makes them suitable for online applications (for a review see Chavarriaga et al., 2014; Kumar et al., 2019b).

The use of error-related potentials as learning signal for reinforcement learning setups brings several advantages to the brain-machine interface setup. First of all, these error signals are naturally generated in the human brain upon error occurrence, without requiring an explicit action from the subject and without imposing any extra mental workload (Kim et al., 2020), which could also make the systems scalable without increasing complexity for the participants (Iturrate et al., 2013a). Moreover, an ErrP can also be present when defining a reward function is not easy or correct behavior is relative (Iturrate et al., 2010a). Finally, as the error is an intrinsic brain signal, it carries subject-specific information that can move brain-machine interface (BMI) development toward its personalized operation (Iturrate et al., 2010a).

Hence, the purpose of this work is to provide the reader with a summary of the available studies that applied error-related potentials in a reinforcement learning-based framework, focusing on the use of ErrP as learning signals. The wide range of work published that integrates error-related potentials in their interfaces demonstrates the feasibility of extracting useful information from these signals. This review focuses on non-invasive brain-computer/brain-machine interfaces that used error-related potentials as learning signals in a reinforcement learning framework. We aim at providing an overview of the current state of development in this field and hope to contribute to discovering unexplored possibilities, bringing the research topic forward.

Chavarriaga et al. (2014) first introduced a 10-year collection of works incorporating ErrPs in brain-computer interface (BCI) systems. More recently, a review focused on brain-computer interfaces for rehabilitation of motor-impaired patients was done by Kumar et al. (2019b). The present work proposes to extend the information collected in these works while exclusively focusing on reinforcement learning-based applications of error-related potentials. The motivation for this summary is that different studies have already successfully shown the advantages of using the ErrP information to undo or correct mistakes made by the interface. However, future work should focus on task learning and ready-to-use systems that do not require long calibration phases and adapt to changes.

The organization of this work is as follows: Section 2 gives a review of the error-related potential phenomenon, with a short description of the different ErrPs reported in the literature. In sequence, we present the reinforcement learning framework in Section 3. Sections 4 and 5 shortly cover BMI applications using ErrPs. Section 6 brings details on the current body of works combining ErrPs and reinforcement learning. Finally, we conclude this work with a brief overview and discussion in Section 7.

## 2. Error-Related Potentials and Their Taxonomy

It was in the 1960s when the first study using implanted electrodes reported evidence of an error-processing system in the brain (Bechtereva and Gretchin, 1969). However, research on error processing significantly increased only in the early 1990s, when studies (Falkenstein et al., 1991; Gehring et al., 1993) using non-invasive EEG devices showed that the occurrence of errors when subjects performed a choice reaction task elicited a specific event-related potential (ERP) in the brain. This ERP signal expressed a fronto-central negative peak that appeared around 50–200 ms after the incorrect response and was named *error-related negativity* (ERN or Ne) (Falkenstein et al., 2000). A centro-parietal positive (Pe) deflection around 200–500 ms after response onset usually follows the ERN (Falkenstein et al., 1991, 2000). Its functional significance remains unclear, but this positivity seems related to the subject's awareness of the error (Falkenstein et al., 2000; Wessel et al., 2011), and some works have also suggested that it shares features with the P300 component measured in oddball paradigms (Ullsperger et al., 2014). This component might, however, not be present in every error trial (Wessel et al., 2011). A positive peak around -50 to 0 ms before response onset can also precede the ERN (Ullsperger et al., 2014).

Miltner et al. (1997) later on described a component similar to the ERN, referring to an ERP signal characterized by a negative deflection appearing over medial-frontal scalp regions around 250 ms after the presentation of a feedback stimulus. Their experiment consisted of a time-estimation task in which subjects were supposed to produce a 1-s interval and signal it *via* a button press. The feedback stimulus informed the subject about the outcome of the performed choice, and the generated ERP component was, therefore, named *feedback-related negativity* (FRN) (or sometimes feedback-ERN or feedback negativity) (Cohen et al., 2007; Ullsperger et al., 2014).

Another extensively investigated ERP component is the N2, prominent negativity that occurs around 200–300 ms after conflicting stimulus presentation. This stimulus-locked component is common in protocols with stimulus mismatch and has been associated with performance monitoring and cognitive control (Yeung et al., 2004; Ullsperger et al., 2014). Often seen together with the P300 component, e.g., in the oddball paradigm, they are still often called the N2-P3 complex (but they can appear independently of each other). In the context of ErrP-based BMIs, these components are also reported as being part of the measured error-related potential (Ehrlich and Cheng, 2016).

The origin of all these components (ERN/FRN/N2) is believed to be the anterior cingulate cortex (ACC) with expected increased modulations in theta frequency (Ullsperger et al., 2014), though some works also report activity in delta or even alpha range (see Yeung et al., 2004; Folstein and Van Petten, 2007 for a review). Different theories such as the mismatch theory, the response conflict monitoring, and the reinforcement learning theory of error processing have tried to explain the ERN, Pe, FRN, N2, and P3 components related to performance monitoring and error processing in the human brain. They aim at providing insight into the functional meaning of each ERP component. To analyze these theories is beyond the scope of this work. Therefore, for details, we recommend the reviews from, e.g., (Falkenstein et al., 2000; Yeung et al., 2004; Walsh and Anderson, 2012; Ullsperger et al., 2014).

In BMI systems, the term ErrPs commonly summarizes the effects of these prominent event-related potential components during performance monitoring, and the difference between error and correct grand averages (averages over all subjects and trials) is used to report the measured ErrPs (Chavarriaga et al., 2014; Ehrlich and Cheng, 2016). This error-minus-correct waveform is applied to isolate the differences between error- and correct-related ERP signals, leaving in the corresponding ErrP wave only those components specifically related to the differences in processing the correct and erroneous events. The measured ErrPs are then characterized by the ERP components found in the difference grand average. One could argue whether every peak in the resulting difference waveshape should be associated with error processing. Indeed, the still not fully understood functional meaning and relationship of the ERP components support that one should be careful with conclusions. Nevertheless, we point out that ErrP-based BMIs works adopt this approach. Most likely because, for the proposed applications, the exact origin and meaning of the difference wave components are irrelevant.

Several works have shown that different experimental paradigms using BMI systems can elicit ErrPs. Seven different ErrPs are commonly mentioned in the existing literature applying non-invasive BMI systems. In sequence, we present a short overview of each one of them.

Self-made errors generated when the subject has to respond as fast as possible to a stimulus are commonly called *response errors*. Attention (Blankertz et al., 2002) tasks, variations of the Eriksen Flanker paradigm (van Schie et al., 2004; Penaloza et al., 2014; Padrao et al., 2016), and the Go/NoGo paradigm (Wirth et al., 2019) are commonly used to generate response errors, which are characterized by the ERN/Pe components in the response-locked ERP (Falkenstein et al., 1991; Gehring et al., 1993).

Similarly, the error-related activity recorded when feedback informs the subject about the outcome of their choice is referred to, specifically in the BMI literature, as *feedback error*. As the response error, it is characterized by errors made by the subject and exhibits the FRN component, sometimes followed by a positivity (Miltner et al., 1997; Lopez-Larraz et al., 2010; Chavarriaga et al., 2014). This error is more commonly studied outside the context of BMIs, using paradigms applying either the time estimation, gambling, guessing games, or reinforcement learning tasks (Ullsperger et al., 2014). In such cases, researchers are interested in understanding the mechanisms of outcome evaluation processes in the brain, and the term FRN is commonly applied (instead of feedback ErrP).

Apart from these two, according to Diedrichsen (2005), a so-called *target error* should be elicited when unexpected changes happen in the task being performed. Typically a reaching (aiming) task is implemented as an experimental setup, and changes in the task are realized by performing a target jump at unexpected moments, such that the subject fails to reach the target object (Diedrichsen, 2005; Krigolson et al., 2008; Milekovic et al., 2013). Target errors are mediated within the posterior parietal cortex and seem to generate a P300 component (relative to movement onset) at the Pz electrode (Krigolson et al., 2008). The P300 component has been associated with context updating and learning.

A widely applied error in the context of BMIs is the *interaction error*, which was first reported by Ferrez and Millan (2007). Defined as the error expected when the subject gives a command and the system executes another, it was measured using a 1D cursor control with a keyboard when the cursor moved in the opposite direction than commanded. Particular to this error is that the erroneous event itself does not come from the subject but the interface misinterpreting the subject's intention. The detected error exhibited a fronto-central activity with a first positive peak 200 ms after feedback onset, immediately followed by a negative and a positive peak around 250 and 320 ms, respectively. Lastly, a broader negative component around 450 ms followed (Ferrez and Millan, 2008a).

**Figure 1:**
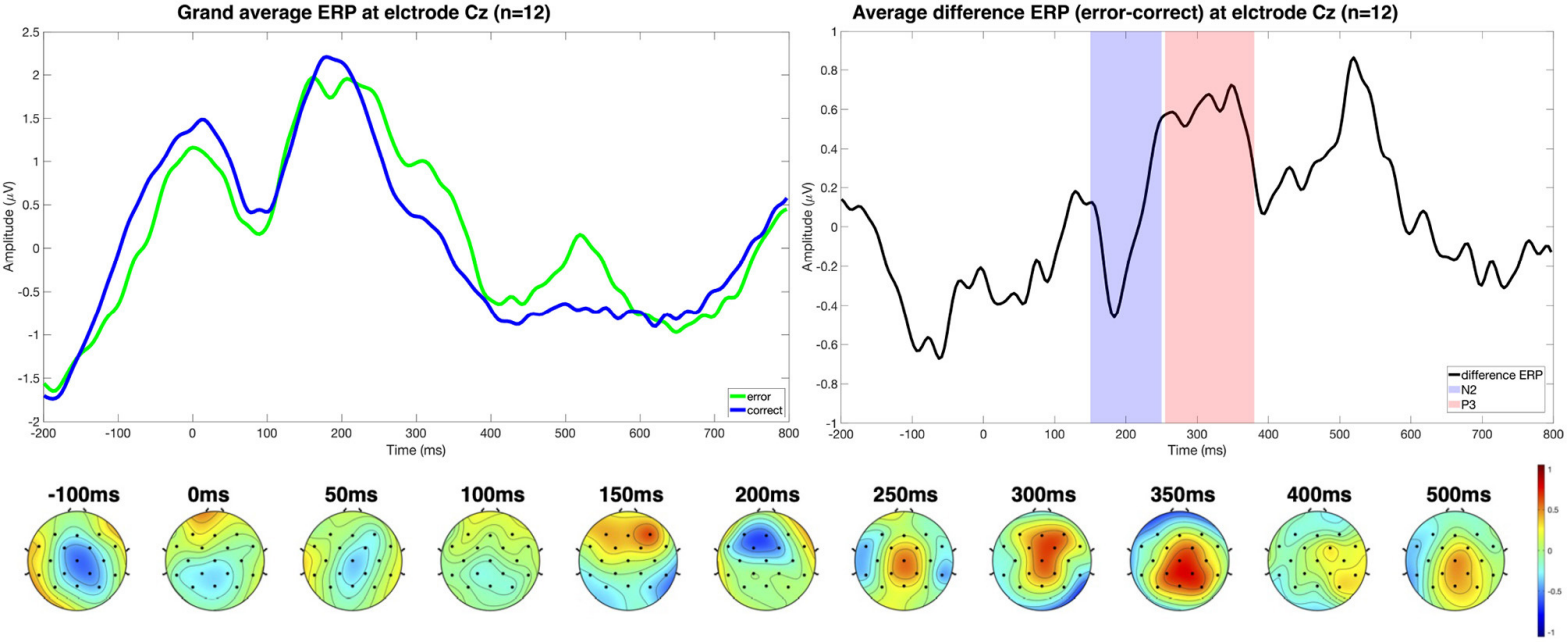
The upper left plot shows the grand average ERPs for each trial class (error/correct) at electrode Cz (*n* = 12). The experimental setup considered a human-robot interaction scenario in which the subject had to respond to a stimulus with a keypress (left, right, or up) indicating the target position, and a real robot turned its head either in the given direction or not, eliciting interaction ErrPs. On the right side, the different ERP displays the N2-P3 complex measured. The topographical distribution of the difference ERP shows that the N2 component is fronto-centrally located, and the characteristic P3 is also centrally located at around 300 ms. Results were reproduced from Ehrlich and Cheng (2016) using the publicly available datasets Ehrlich and Cheng (2019).

**Figure 2:**
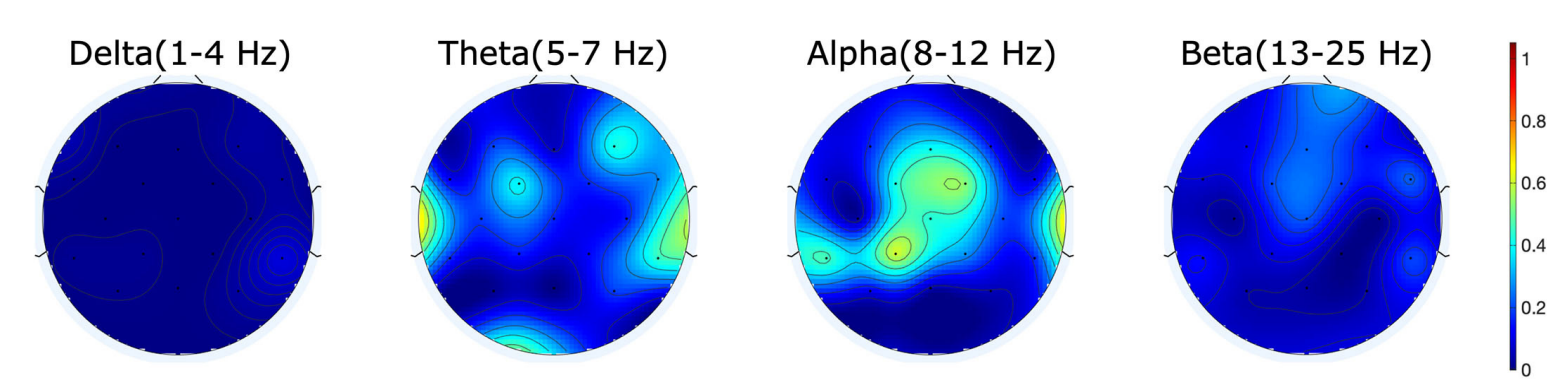
*R*^2^-values showing the difference in power for the different frequency bands between correct and error trials show activity mainly in the alpha range for the measured error.

Other works have evaluated the interaction error under different experimental setups as well, including cursor control together with motor imagery (Ferrez and Millan, 2008b, 2009), BCI spellers (Margaux et al., 2012; Bevilacqua et al., 2020), and simulated control of a car (Zhang et al., 2013). Figures 1, 2 show, for example, the interaction error reported in a human-robot interaction task by Ehrlich and Cheng (2016). The authors specifically highlight the N2-P3 complex is present in the difference grand average (error minus correct trials).

A similar error named *execution error*, expected when the current motor commands result in unexpected movement was defined by Diedrichsen (2005). Spüler et al. (2015) have defined an execution error as when the cursor in a game goes in a different direction than the one received from the joystick controller during continuous feedback. Modifications in the cursor movement degree generated erroneous feedback that triggered execution errors. From our perspective, this last definition does not seem far from the setup used by Ferrez and Millan (2008a) to elicit interaction errors. In both cases, the system executed something other than the command given. Therefore, one could argue that these errors could be the same, despite the two names used. We do not want to infer that those errors are the same thing. In fact, from our understanding, one cannot exclude the possibility that the confusion arises from a thin line between the definitions of these errors in specific scenarios. This fact does not necessarily mean that they are always the same. But we considered it relevant to mention and catch attention for these interesting similarities or, at least, apparently ambiguous situations. In addition, execution and interaction errors showed similar waveshape and topographical distributions. The execution error collected showed a first positive peak around 229 ms followed by a negative peak around 287 ms that, according to Spüler and Niethammer (2015), is possibly related to the FRN. A positive peak around 367 ms followed this negativity, which the authors associate with Pe. Finally, a broader negative deflection around 461 ms was related to the N400. These components were maximum at fronto-central electrodes FCz and Cz. Unlike interaction errors, however, execution ErrPs have been often used under continuous feedback (Lopes Dias et al., 2018, and involving misalignment or displacement (Rakshit et al., 2016; Lopes-Dias et al., 2019).

Several works reported the execution error in parallel to another error: the *outcome error*, expected when the desired goal of a movement is not fulfilled (Krigolson et al., 2008). Outcome errors are also present in the cursor game, in which the subject controls the cursor *via* keypress or joystick, and collisions with falling blocks elicit outcome errors (Spüler and Niethammer, 2015; Spüler et al., 2015). The authors have reported an outcome error characterized by a negative component at 2 ms, which they say is the ERN, followed by a positive peak around 268 ms (the Pe, according to them). A negative peak at 486 ms (N400) and a small positivity at 742 ms follow. These components were maximum at fronto-central electrodes. Kreilinger et al. (2016) have applied a similar approach using a car game where a collision could happen with coins (correct) or obstacles (error). However, they have only observed a positivity around 200 ms followed by a negative peak around 400 ms after collision onset at Fz and Cz channels.

Last but not least, we have the by far most explored type of error-related potentials in non-invasive BMI systems: the *observation error*. In van Schie et al. (2004), authors have shown that the same mechanisms for error processing in the brain are active in response to errors committed by others, providing the first evidence of this error. Using a modified Eriksen flanker task, they measured an ERN peaking at 252 ms after response onset. Since the latency, the scalp distribution, and the source of this negativity resembles the ERN, this component is sometimes also called observation ERN (oERN) (Ullsperger et al., 2014).

Given its implementation simplicity, the observation error is widely used in BMI frameworks. Several studies have shown that this type of error is elicited when the subject realizes an error made by an external system over which they have no control in different applications. Across these works, observation errors are reported with different characteristic components. The observed ErrP showed positive and negative peaks at 200 ms and 260 ms, respectively, followed by an additional positivity at 330 ms in a cursor observation task (Chavarriaga et al., 2007; Chavarriaga and Millan, 2010). On the other hand, only a positivity at 300 ms followed by a negativity at 400 ms was found during robot performance observation, with both simulated (Iturrate et al., 2010a) and real robots (Iturrate et al., 2010b).

The different ErrPs and works listed in this section are summarized in Table 1. Each work reports different peaks and latencies, even when using similar experimental tasks. Therefore, it is not possible to conclude that an ErrP will always exhibit the waveshape as originally reported. We highlight this because we have asked ourselves whether we could define which components to expect in each ErrP type mentioned in the literature. After reviewing the several works we list here, we did not converge to a unique ErrP type-component specification. Therefore, we restrict ourselves to indicating some reported components across the reviewed works, such that it can help build a first impression of the respective ErrP. We believe it is especially relevant when analyzing data from new experimental protocols to have a baseline for comparison. Otherwise, any measured grand average might be wrongly associated with an ErrP.

**Table 1:** Summary of the error-related potentials defined in Section 2.

**Error**	**Task**	**Stimulus**	**#subjects**	**Error rate (%)**	**Electrodes**	**Components**	**Time-locking event**	**References**
Response	Choice reaction Go/NoGo Eriksen flanker	Visual auditory	91 healthy	5−25	Fz, FCz, Cz, Pz	Neg. 80 ms (Ne), pos. [200-500] ms (Pe)	Response onset	Falkenstein et al., 2000
	Modified Eriksen flanker	Visual	6 healthy	n.a.	Cz	Neg. 100 ms (ERN)		Gehring et al., 1993
			18 healthy	7.9		Neg. 80 ms (ERN)		van Schie et al., 2004
Feedback	Time estimation	Visual auditory somatosensory	18 healthy	50	FPz, Fz, Cz, Pz	Neg. [230–330] ms (FRN)	Feedback onset	Miltner et al., 1997
		Visual	5 healthy	50	FCz	Neg. 320*ms*, pos. 430 ms, Neg. 530 ms		Lopez-Larraz et al., 2010
Target	Manual aiming	Visual	15 healthy	n.a.	Pz	Pos. 341 ms (P300)	Target jump onset	Krigolson et al., 2008
Interaction	Cursor control	Visual	3 healthy	20	Cz	Neg. 270 ms (Ne), pos. [350-450] ms (Pe), Neg. 550 ms	Feedback onset	Ferrez and Millan, 2007
			5 healthy	20, 50	FCz	Pos. 200 ms, Neg. 250 ms, pos. 320 ms, Neg. 450 ms		Ferrez and Millan, 2008a
			2 healthy	20		Pos. 200 ms, Neg. 270 ms, pos. 300 ms, Neg. 430 ms		Ferrez and Millan, 2008b
			6 healthy			Pos. 230 ms, Neg. 290 ms, pos. 350 ms, Neg. 470 ms		Ferrez and Millan, 2009
	Robot control		13 healthy	35	Cz	Neg. 200 ms (N2), pos. 300 ms (P3)		Ehrlich and Cheng, 2016
	BCI speller		16 healthy	38	Cz	Neg. 350 ms, pos 480 ms		Margaux et al., 2012
		Visual, none	10 healthy	20	CPz	Neg. 250 ms, pos. 500 ms		Bevilacqua et al., 2020
	Simulated car	Visual	7 healthy	30	FCz, Cz	Neg. 250 ms, pos.400 ms		Zhang et al., 2013
Execution	Cursor control	Visual	10 healthy	55	FCz	Pos. 229 ms, Neg. 287 ms (FRN), pos. 367 ms (Pe), Neg. 461 ms (N400)	Error onset	Spüler and Niethammer, 2015
			15 healthy	30		Neg. 246 ms, pos. 354 ms, Neg. 568 ms		Lopes-Dias et al., 2019
		Visual normal Visual jittered	15 healthy	30		Neg. 196 ms, pos. 404 ms, Neg. 616 ms		Lopes Dias et al., 2018
	Robot control	Visual	5 healthy	n.a.	Fz	Neg.150 ms, pos.[300-500] ms		Rakshit et al., 2016
Outcome	Cursor control	Visual	15 healthy	n.a.	Cz	Neg. 268 ms (ERN)	Movement end	Krigolson et al., 2008
			10 healthy	15	FCz	Neg. 2 ms (ERN), pos. 268 ms (Pe), Neg. 486 ms (N400), pos. 742 ms		Spüler and Niethammer, 2015
	Car game	Visual auditory, none	10 healthy	22.7	Fz, Cz, Pz	Pos. 200 ms, Neg. [400-500*ms*] ms	Collision onset	Kreilinger et al., 2016
Observation	Modified Eriksen flanker	Visual	18 healthy	9.1	Cz	Neg. 252 ms (ERN)	Response onset	van Schie et al., 2004
	Cursor control		3 healthy	20	FCz	Pos. 200 ms, Neg. 250 ms, pos. 350 ms, Neg. 500 ms	Feedback onset	Chavarriaga et al., 2007
			6 healthy	20, 40		Pos. 200 ms, Neg. 260 ms, pos. 330 ms		Chavarriaga and Millan, 2010
	Robot control		2 healthy	80		Pos. 300 ms, Neg. 400 ms		Iturrate et al., 2010a
			4 healthy					Iturrate et al., 2010b

*Please note that this table does not include an exhaustive list of available works. The ErrP components are presented in terms of the reported peak/latencies and the component name given by the authors, when applicable. Values marked gray were inferred from available information, and n.a. indicates not available*.

## 3. Reinforcement Learning

The idea of learning from interaction has supported theories on how we humans learn and how what we understand as intelligence is defined (Sutton and Barto, 2018). Comprehending the learning processes in humans and animals has been the focus of different theories in psychology studies, e.g., in the law of effect learning (Thorndike, 1898), conditional (Pavlovian) learning, and instrumental (operant) conditioning (Skinner, 1965). Sutton and Barto (2018) developed the concept of reinforcement learning as a computational approach to learning from interaction. Their book presents the theory that supports an uncountable number of works available in the literature that involve reinforcement learning approaches, including the ones summarized here. Therefore, for a deeper understanding of the theory and concepts of reinforcement learning, we forward the interested—and especially new readers—to this relevant book. Additionally, we reinforce that the definitions presented in this section have the book as the principal source, and we focus on the fundamental components of a classic reinforcement learning framework.

**Figure 3:**
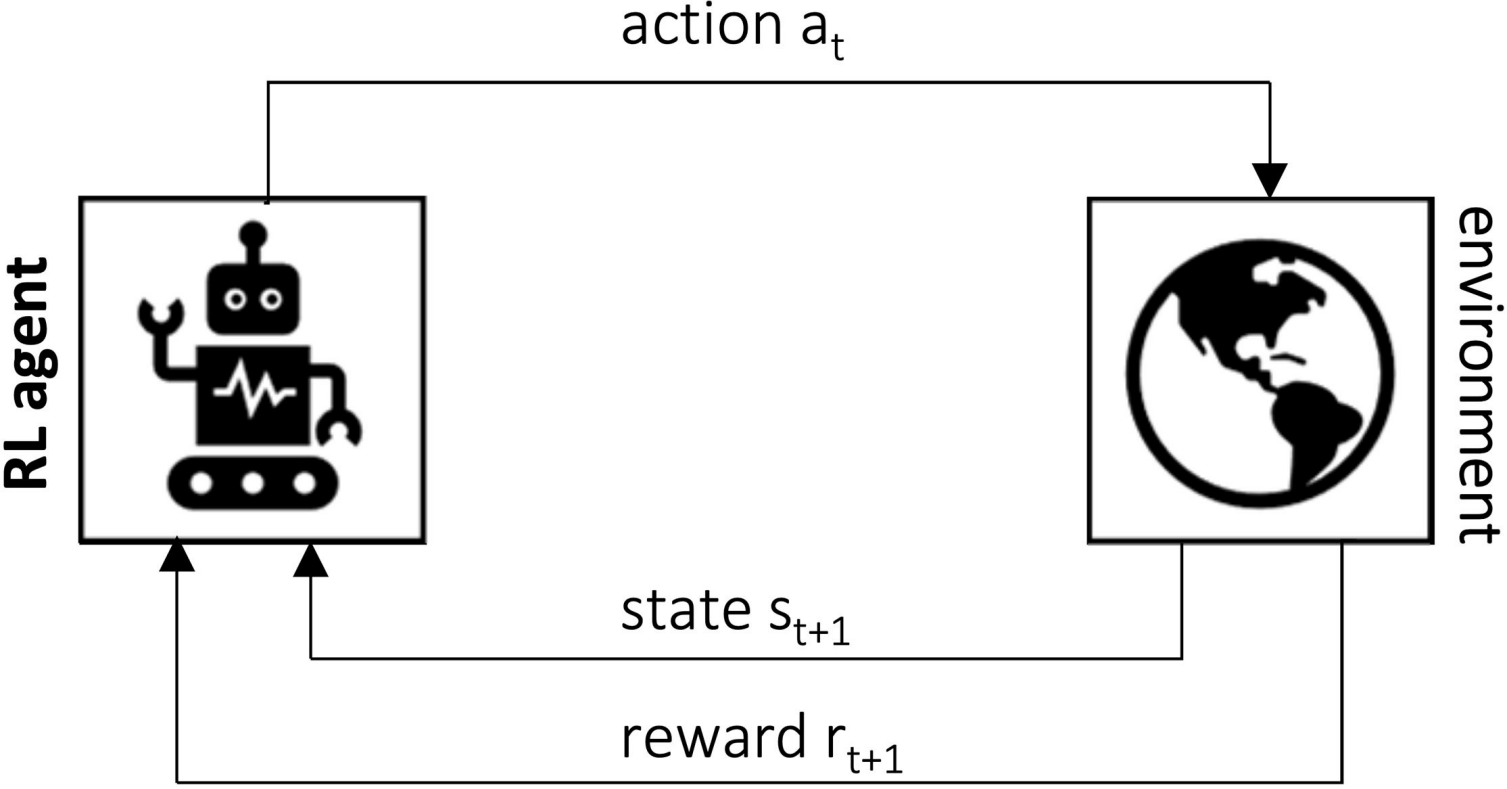
The agent-environment interaction process in an RL setup (Sutton and Barto, 2018).

Reinforcement Learning can be defined as learning how to map situations to actions in order to maximize a numerical reward signal (Sutton and Barto, 2018). To achieve its task, the learner—here called agent—must use a trial-and-error approach, since no information is given about which actions should be taken in each step. For each action taken, the agent receives an immediate reward that quantifies its choice, and its goal in the reinforcement learning problem is to maximize the total reward accumulated. The agent interacts with the environment in a sequence of discrete time steps *t* = 0, 1, 2, …. At each time step *t*, based on the information it receives from the environment regarding its state s∈S, it takes an action a∈A(s). Then, one time step later, as a consequence of the action chosen, it receives a *reward* $r_{t+1}\in R$ and moves to a new state *s*_*t*+1_. This interactive process is shown in Figure 3.

One challenge arising in RL that is not present in the other types of machine learning is the so-called exploration-exploitation trade-off. As time passes, the agent learns to exploit the information collected about which actions generate higher rewards. On the other hand, without the information about which actions are the good ones, it has to explore its possibilities to find them (Sutton and Barto, 2018). Additionally, in more complicated cases, the chosen action might not only affect the immediate reward received but also the next situation and, consequently, future rewards. Together, the trial-and-error approach and the delayed reward problem form the core characteristics of reinforcement learning (Sutton and Barto, 2018).

Formally, RL handles the solution of a Markov Decision Process (MDP). The MDP framework is widely used as a classical way to formalize sequential decision-making in scenarios involving delayed rewards (Silver, 2015; Sutton and Barto, 2018). It proposes that it is possible to reduce any problem of learning goal-directed behavior to three signals exchanged between agent and environment: state, action, and reward. The state carries information about the environment status, but what it represents depends on the problem. In simpler cases, it can be a finite discrete set. However, more complex scenarios usually involve continuous states. The same happens with the actions the agent can perform. Together, they increase problem complexity. The reward signal models the goal of the reinforcement learning task, and the design of a meaningful reward function is not a trivial task. In general, it is important to know that these three basic components vary among tasks, and their definitions have a direct impact on learning performance.

The learning task itself is goal-oriented and aims at learning an optimal policy that defines the agent's behavior. The policy specifies which action should be taken in each state and therefore directly influences the received reward. An optimal policy is a policy that leads to the highest return in the long run, and more than one may exist. This optimal policy can be determined *via* value functions that estimate how good it is for the agent to be at a specific state (state-value function) or how good it is for the agent to take a specific action at a certain state (action-value function). This estimation can be modeled as an iterative process, and both the value function and the policy will converge to their optimal values by the end of these interactions (for details see Sutton and Barto, 2018). Therefore, many reinforcement learning methods follow this formulation.

These methods can be categorized according to their approach to finding this optimal policy. If a model of the environment is available, dynamic programming methods can be applied. Otherwise, Monte Carlo or Temporal Difference ones can be chosen. One can also distinguish between on-policy methods, which use samples collected while acting according to the current policy under evaluation, and off-policy methods that evaluate one policy while acting based on another. The most popular example of off-policy temporal difference methods is Q-learning, which directly learns the optimal value function. Alternatively, instead of learning the value functions, it is also possible to directly search for the optimal policy, which is more suitable for high-dimensional or continuous action spaces (Silver, 2015). Finally, the so-called actor-critic methods consider the advantages of both value-based and policy-based methods and combine them by learning both the value function and the policy simultaneously.

## 4. Error-Related Potentials in Brain-Machine Interfaces

Error-related potentials have been investigated under different experimental setups using non-invasive BMIs. Several of these works have focused on detecting such signals under the given conditions. In such cases, the focus is on demonstrating that ErrPs can be elicited using the proposed experimental paradigm. As discussed in Section 2, ErrPs are generated in the brain not only when the subject realizes a self-made error but also during interaction or observation of an external system operation.

Self-made errors have been widely studied using attention (Blankertz et al., 2002) and choice reaction tasks applying for example the go/no-go (Wirth et al., 2019) or Eriksen flanker paradigms (Gehring et al., 1993; Falkenstein et al., 2000; Padrao et al., 2016). Chavarriaga et al. (2007); Iturrate et al. (2010b) and Yousefi et al. (2018) have shown that ErrPs are elicited when the subject has to engage in tasks that require a high level of concentration (e.g., motor imagery or cognitive task). The presence of an ErrP when the subjects evaluate the actions of a robotic arm based only on individual subjective criteria was addressed by Iwane et al. (2019). The possibility to detect ErrP in a virtual reality environment has been evaluated by Si-Mohammed (2020); Padrao et al. (2016); Singh et al. (2018); Gehrke et al. (2019) and with projection systems in Chavarriaga et al. (2012); Pavone et al. (2016); Yazmir and Reiner (2017); Pezzetta et al. (2018). Lastly, Kumar et al. (2019a) have investigated if an ErrP signal is evoked when stroke patients are unable to perform a physical exercise. Since our goal is not to provide a review of the detection of ErrP, these are just some in a long list of available works that have only considered error detection. For additional information, we recommend seeing Chavarriaga et al. (2014) and Kumar et al. (2019b) for other references.

Fewer works have gone further and proposed using these error signals instead of just detecting them. Combaz et al. (2010); Chavarriaga et al. (2010) and Zhang et al. (2018) have introduced theoretical analysis of the potential benefits of applying an online ErrPs detection to improve BMIs performance. The first systems using the detected ErrPs were presented in Parra et al. (2003) to correct human response errors based on the detected ERN and in Ferrez and Millan (2008b) to stop cursor movement.

With more popularity, ErrPs have been extensively applied to BCI spellers. Dal Seno et al. (2010) have shown the first attempt at using a P300 BCI speller with an integrated error-correction mechanism based on ErrPs, by canceling character selection upon error detection. However, they measured no improvement in their interface and attributed this to the low ErrPs classification accuracy. With a similar approach, Schmidt et al. (2012) have reported that false-positives in ErrPs classifier output influenced overall system performance. Margaux et al. (2012) and Cruz et al. (2018) have additionally proposed automatically replacing characters with the second-best letter. Chavarriaga et al. (2016) have presented a novel BCI speller without the P300 component in which the cursor moves in a matrix toward the most probable character as inferred based on a language model and the decoded ErrPs.

In the position control of a robotic arm, ErrPs have been used as a feedback response to undo action when the robot moved in the wrong direction (Bhattacharyya et al., 2014), to change to the other possible direction (Salazar-Gomez et al., 2017), as well as to compensate offset errors at end position (Bhattacharyya et al., 2014, 2017; Rakshit et al., 2016). In other applications, the system replaced the wrong selection with the second-best alternative (Penaloza et al., 2014). In gesture-based BCIs, Putze et al. (2015) have used error signals in different ways: to only undo a wrong gesture, to select the second-best one, or to wait for the user to perform a manual correction gesture.

Lastly, in the rehabilitation context, Rotermund et al. (2006) have proposed an online adaptation scheme to control a prosthetic arm. However, they provided only simulation results using a hypothetical error signal. More recent work has analyzed the application of ErrPs as a feedback signal to cancel a command sent to a lower-limb exoskeleton (Zhang et al., 2018). However, they do not seem to have applied their idea to a real exoskeleton. Perrin et al. (2010) have introduced a novel semi-autonomous navigation strategy for an intelligent wheelchair. Their system recursively proposes an action until the user accepts it.

These are only some of the available works that have proposed using the ErrPs information to improve their systems by applying an error correction strategy. For an overview, please see Supplementary Table 1.

## 5. Error-Related Potentials-Based Learning

**Figure 4:**
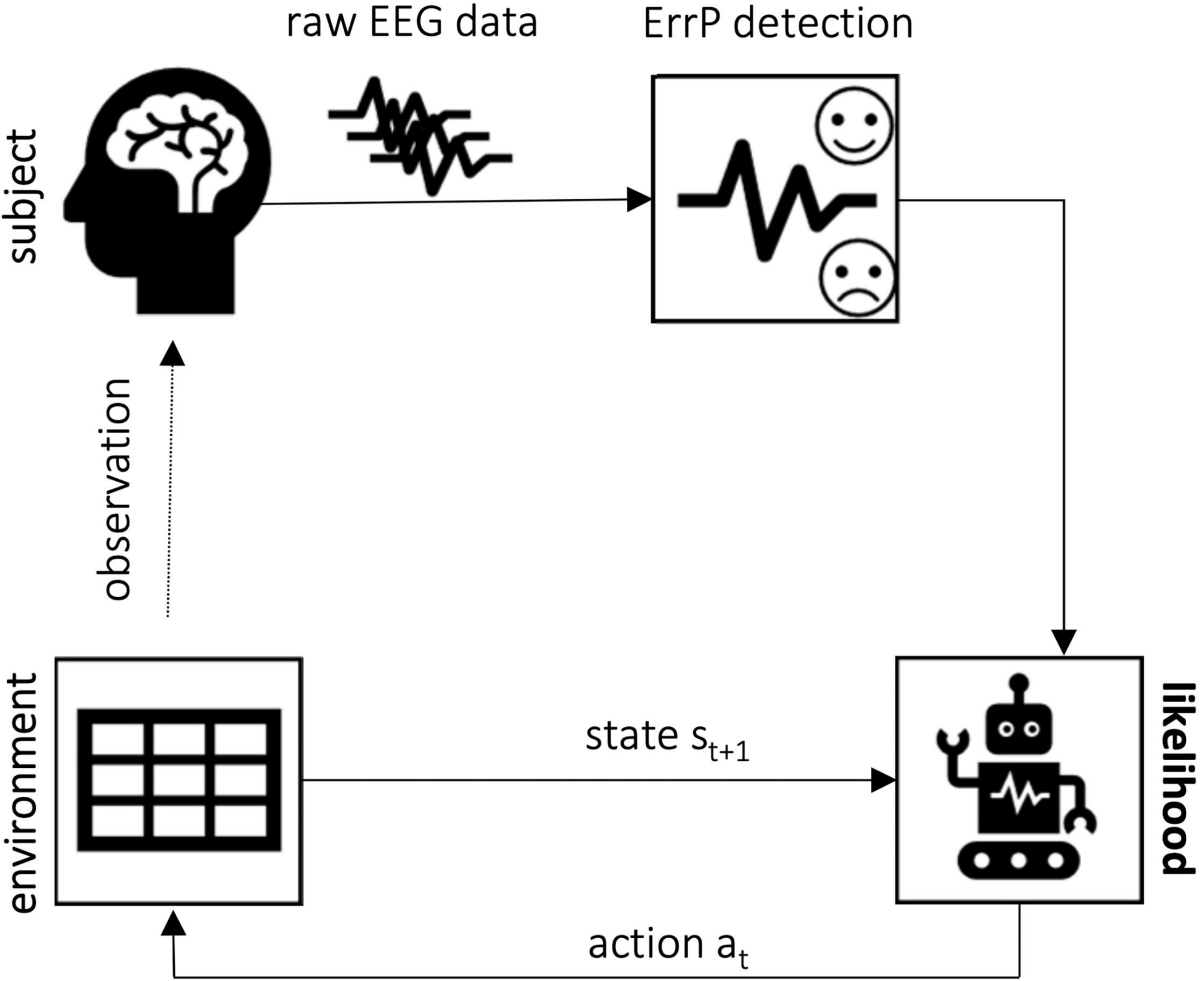
Error-based learning framework concept: upon EEG data classification, the error information can be used to update the likelihood of, for example, performing each action, decreasing it upon error detected or increasing, otherwise. This approach has been used by Chavarriaga et al. (2007, 2010). Iturrate et al. (2015b), on the other hand, have used the error information to update the likelihood of each possible position being the desired goal.

In this section, we summarize the works that have explored using ErrPs as learning signals but that have not applied a reinforcement learning framework as defined in Section 3. The learning approach applied is shown in Figure 4 and consists of using the ErrP information to update probability values. An overview is also given in Table 2. A flow diagram of the review process applied in this work to define the studies to report in this and in Section 6 can be found in Supplementary Figure 1.

**Table 2:** Overview of brain-machine interface systems that use error-related potential-based learning frameworks.

**Application**	**Task**	**References**	**#subjects**	**User task**	**Learning task**	**ErrP signal**	**Reward function**	**State space**	**Action space**	**Learning algorithm**
Cursor control	1D reaching task	Chavarriaga et al., 2007	3 healthy	Observe cursor	Learn user's optimal policy to reach target (offline)	Observation ErrP	-	1 target position	2 actions [left, right]	Likelihood
	1D reaching task	Chavarriaga and Millan, 2010	6 healthy	Observe cursor	Learn user's optimal policy to reach target (offline)	Observation ErrP	-	1 target position	2 actions [left, right]	Likelihood
	2D reaching task	Iturrate et al., 2015b	8 healthy	Observe cursor	Learn user's chosen target and optimal policy to reach it and simultaneously train ErrP classifier (online)	Observation ErrP	$R=\{\begin{array}{cc} 0 & \text{if target is reached}\\ +1 & \text{otherwise} \end{array}$	25 grid positions	5 actions [left, right, up, down, reach]	Likelihood
Robot control	2D reaching task	Iturrate et al., 2013b	1 healthy	Observe mobile robot	Learn user's chosen target and intended strategy to reach it (online)	Observation ErrP	-	3 values [position, orientation]	2 actions [rotation, linear movement]	Likelihood

To provide the reader with an overview of what has been done in the field we present the experimental setup employed in each work together with the task to be learned based on the error information. An additional focus lies on how the error information has been applied. For that, we describe the learning framework used and how each work has included the error information. We highlight existing similarities (e.g., regarding the learning task) among these works and points that have been further investigated. To demonstrate to what extent the proposed approaches have been evaluated, we emphasize the total number of subjects and the experiments performed. We also considered it relevant to provide details on the measured ErrP. By mentioning the component-latency information and the electrode used, we hope to help build a first impression of the respective ErrP shape. Finally, further tests and comparisons associated with error decoding or learning performance are also reported.

A first attempt to use the detected ErrPs to improve system performance by learning how to commit fewer errors in the future has been proposed by Chavarriaga et al. (2007). Their approach considered a 1D cursor control task with three fixed positions: two possible targets and one current cursor position. The subject had to observe and evaluate the system performance considering that the task goal was to bring the cursor to the highlighted target position. At each timestep, the cursor moved toward or away from the target. The cursor followed a sub-optimal control policy with an error rate of 20%.

Experiments with three subjects showed that an observation ErrP could be measured and reliably detected on a single-trial basis with an average accuracy of 76%. The measured error showed the characteristic peaks at channel FCz (pos. 200 ms, neg. 250 ms, pos. 350 ms, neg. 500 ms, latencies approximated from the provided plot) and was applied in a reinforcement learning alike framework. The optimal control strategy to be learned was expressed in terms of probabilities, and the ErrPs were used to update the likelihood of performing each action given the target location. Essentially, upon error detection, the probability of performing action *A*_*t*_ at time *t* given target location *T*_*t*_ was decreased. Offline learning analysis showed that the optimal control policy was learned in about 40 trials. This same approach has been further evaluated with six subjects in a larger working space with twenty possible cursor positions and random targets (Chavarriaga and Millan, 2010). A similar learning performance has been observed, with the probability of performing the correct action converging to 1 after 50 trials. However, once again, only offline analysis has been performed. In this experiment, the error grand average only displayed three prominent peaks (pos. 200 ms, neg. 260 ms, neg. 330 ms). Why the later negativity was not observed in this case has not been investigated. How and why such small changes in the experimental protocol can already highly affect the ErrP shape needs clarification.

The 2D cursor control experimental protocol specified by Iturrate et al. (2013a) (described in Section 6) has been further used to create a framework to learn not only the task (i.e., the reaching goal) but also the ErrP decoder itself in a new approach for self-calibration of BCI systems. This is because a requirement for ErrP-based learning applications is the possibility of reliable single-trial-based detection of such signals. Hence, experimental protocols usually start with a calibration phase with trials under controlled error rates (usually around 20–30%). These trials are later on used to train a subject-specific ErrP decoder. However, calibration is a time-consuming task that takes precious minutes. Aware of this limitation, Iturrate et al. (2015b) have shown that it is possible to learn both simultaneously in an unsupervised manner by exploiting known tasks constraints (i.e., the finite number of reaching positions).

By assuming that the user always followed an optimal policy, given a set of all possible goals, based on the observed EEG data, the learning algorithm identified the intended task. The expectation was that actions that agreed with the optimal control policy for the user's current intended task would not elicit ErrP signals. Accordingly, tasks coherent with the measured brain signals received a higher likelihood, and a planning algorithm was applied to choose among these tasks.

After identifying the task with high confidence, the system followed the greedy control policy to reach the goal. In online experiments performed with eight subjects, they measured a characteristic fronto-central ErrP with significantly large positive and negative peaks at 400 and 600 ms, respectively. While comparing with a standard calibration approach, they have found no significant differences in the measured ErrP. Moreover, they have calculated the online decoder accuracy based on ground truth labels and the percentage of correctly identified labels. The overall online decoding accuracy was significantly similar for both calibration approaches, suggesting that the proposed framework did not decrease classification performance.

Using this same supervised calibration method, simulations for all eight subjects using incremental learning resulted in an average ErrP decoding accuracy of 68.4% ± 6.69, with 202 ± 75 trials required for calibration. Decoding accuracy obtained with the self-calibrating approach was significantly similar. However, the ErrP-based control achieved significantly better results in terms of the number of correct trials reached (6.88 vs. 3.97) and the number of steps until the first target (165.25 vs. 305.72), which demonstrates the power of the proposed method. On the other hand, it also reached more incorrect targets (1.50 vs. 0.10). We agree with the authors that this might be because of the low initial accuracy of the classifier. They have additionally suggested that the system's attempt to reduce uncertainty in the signals instead of moving toward the target might have confused the subjects. The decreasing trend observed in error ratio in the last ten trials until reaching the first target provided further evidence that the system learned the task already with the first target. One limitation of this approach is that it relies on the existence of task constraints to generate all possible task outcomes. On the other hand, authors have argued that the proposed approach could be applied as an extension to a supervised method for improved results instead of being a replacement.

## 6. Error-Related Potentials in RL-Based Brain-Machine Interfaces

**Figure 5:**
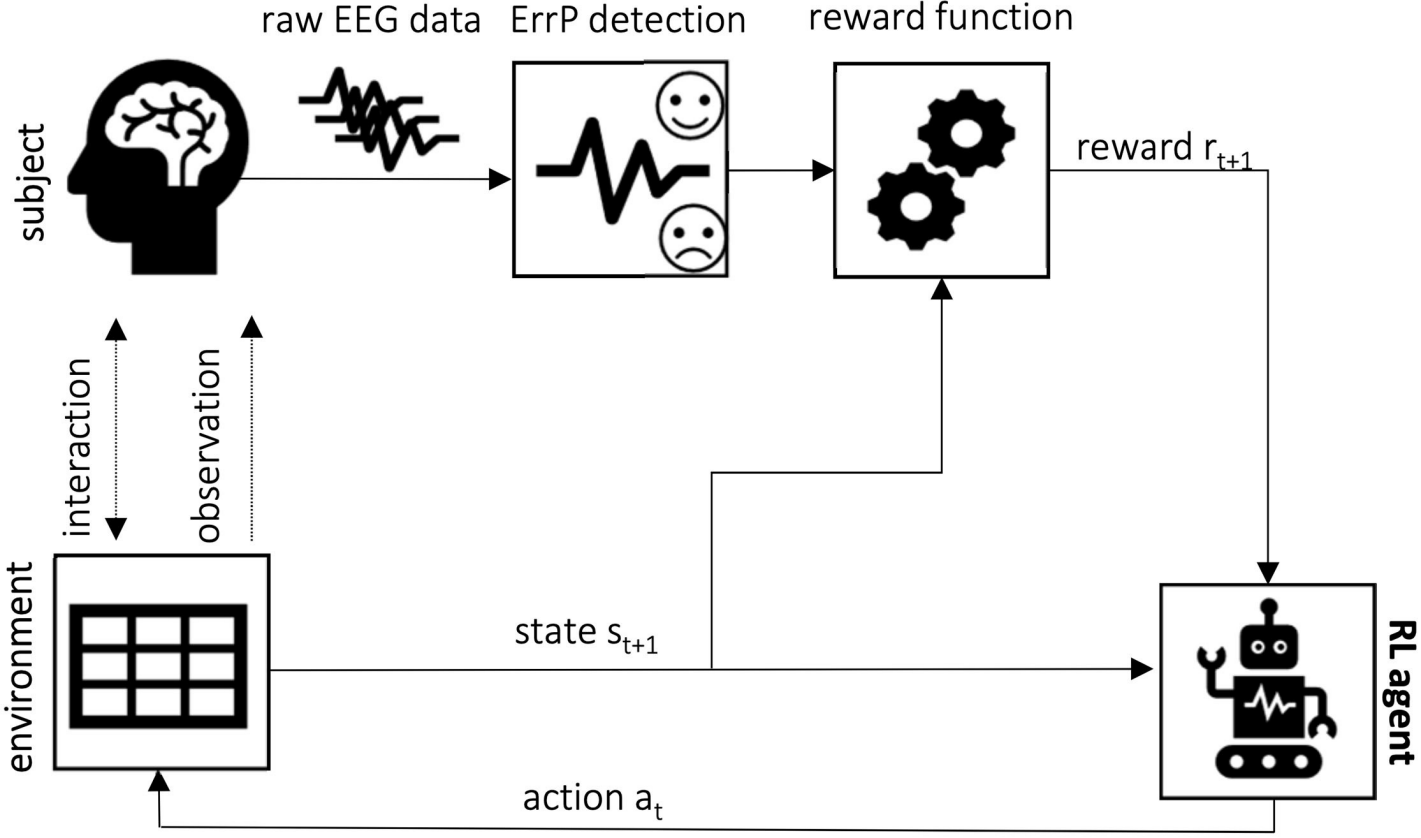
Error-based reinforcement learning framework concept: the ErrP is used as a reward to guide the RL agent as it learns the optimal policy to achieve the desired task. The error-based reward functions penalize wrong actions and reinforce those evaluated as the correct expected behavior by the subject.

The purpose of this review is to summarize the existing literature on the application of ErrPs as learning signals in reinforcement learning-based setups. Therefore, this section describes in more detail works dealing with this topic. For better understanding, we grouped them based on the error applied. The learning approach is mostly based on the use of the ErrP as a reward for the RL agent (Figure 5). An overview is also given in Table 3.

**Table 3:** Overview of brain-machine interface systems that use error-related potential-based reinforcement learning frameworks.

**Application**	**Task**	**References**	**#subjects**	**User task**	**RL task**	**ErrP signal**	**Reward function**	**State space**	**Action space**	**RL agent**
Cursor control	2D reaching task	Iturrate et al., 2013a Iturrate et al., 2013b	4 healthy	Observe cursor	Learn optimal policy to reach target (offline) and learn user's chosen target and reach it (online)	Observation ErrP	Not mentioned	25 grid positions	5 actions [left, right, up, down, reach]	Q-learning and likelihood
	1D reaching task	Iturrate et al., 2015a	12 healthy	Observe cursor	Learn user's chosen target and optimal policy to reach it (online)	Observation ErrP	$R=\{\begin{array}{cc} -1 & \text{if error detected}\\ +1 & \text{if no error} \end{array}$	9 target positions	2 actions [left, right]	Q-learning
Robot control	1D reaching task	Iturrate et al., 2010a	2 healthy	Observe virtual robot	Learn user's chosen target and reach it (online)	Observation ErrP	$R=\{\begin{array}{cc} -1 & \text{if large error}\\ -0.5 & \text{if small error}\\ +1 & \text{if no error} \end{array}$	5 target positions	5 actions [one for each target]	Q-learning
	2D reaching task	Iturrate et al., 2015a	12 healthy	Observe virtual robot observe real robot	Learn user's chosen target and optimal policy to reach it (online)	Observation ErrP	$R=\{\begin{array}{cc} -1 & \text{if error detected}\\ +1 & \text{if no error} \end{array}$	13 grid positions	4 actions [left, right, up, down]	Q-learning
	2D reaching task	Kim et al., 2017	7 healthy	Make gesture and observe robot	Learn to recognize human gestures and map them into robot commands (online)	Observation ErrP interaction ErrP	$R=\{\begin{array}{cc} 0 & \text{if error detected}\\ +1 & \text{if no error} \end{array}$	palm normal vector and grip strength	3 actions [left, right, forward]	LinUCB
	Guessing game	Ehrlich and Cheng, 2018	16 healthy	Observe robot	Adapt gazing policy to subject's guessing policy (online)	Feedback ErrP	$R=\{\begin{array}{cc} -1 & \text{if error detected}\\ +1 & \text{if no error} \end{array}$	4 gazing options [3 objects, human]	4 actions [gazing options]	Policy gradient
	2D reaching task	Schiatti et al., 2018	8 healthy	Observe robot	Learn user's chosen target and intended strategy to reach it (online)	Observation ErrP	$R=\{\begin{array}{cc} 0 & \text{if error detected}\\ +1 & \text{if no error} \end{array}$	25 grid positions	4 actions [left, right, up, down]	Modified Q-learning
	2D reaching task	Kim et al., 2020	8 healthy	Make gesture and observe robot	Learn to recognize human gestures and map them into robot commands (online)	Observation ErrP interaction ErrP	$R=\{\begin{array}{cc} -0.25 & \text{if error detected}\\ +1 & \text{if no error} \end{array}$	palm normal vector and grip strength	4 actions [left, right, forward, upward]	LinUCB
	2D reaching task	Akinola et al., 2020	7 healthy	Observe robot	Learn to navigate in environment with obstacles to reach a target position (online)	Observation ErrP	$R=\{\begin{array}{cc} +100 & \text{if target is reached}\\ -100 & \text{if collision occurs}\\ -1 & \text{otherwise} \end{array}$	13 values [laser range data, target displacement and robot yaw]	3 actions [forward, turn left, turn right]	PPO
	Binary selection	Luo et al., 2018	12 healthy	Observe robot	Learn to solve RL problem (four different problems)	Observation ErrP	Not specified	Not specified	Not specified	Not specified
Rehabilitation device	Open/close hand	Roset et al., 2014	1 healthy 1 SCI patient	Perform hand open/close	Learn to classify motor potentials into device commands (online)	Interaction ErrP	$R=\{\begin{array}{cc} -1 & \text{if error detected}\\ +1 & \text{if no error} \end{array}$	12 normalized PSD z-scores (ErrPs) 50 normalized PSD z-scores (MP)	2 actions [open, close]	Actor-critic

### 6.1. Learning While Observing

Iturrate et al. (2010a) have proposed learning a new task based on the observation ErrP. In their experiments, two subjects observed a virtual robot with two degrees of freedom (DOF) perform a reaching task with five possible positions. To verify the possibility of detecting different types of errors in terms of magnitude and direction, the subjects were instructed to evaluate the system considering that the central target position was the correct one, positions right next to the center were a small error, and targets far from the center should be considered a large error in execution. The measured observation ErrP showed a positivity at 300 ms followed by a negativity at 400 ms. Moreover, they classified the different error directions (left/right: ~90% and >80% for each subject, respectively) and the magnitude (large/small: ~70% and ~60%) with accuracies above the chance level for both subjects.

To demonstrate that the measured error signals can be discriminated and applied in an RL scenario to learn a similar but different task, they have used the standard Q-learning with an ϵ-greedy strategy to learn the optimal policy for determining the correct target. This time, however, the target was freely selected by the subject. The reward for the RL agent was defined based on the different error levels. With a freely chosen target position, the error severity definition changed slightly. However, they have used transfer learning and applied the ErrP-classifier trained previously with pre-defined targets. This approach decreased the detection ratio and affected learning convergence. A problem we believe was accentuated by their reward definition. Therefore, they report the results of 20 executions. For subject 1, 92% of the runs converged to the correct selected target (in around 70 steps), whereas for subject 2, the performance was around 75% (100 steps required). They have not further analyzed why the performance for the second subject was so low. We suppose it is due to the target choice since subject 2 chose the left-most position as the target, and subject 1 chose the position exactly to the right of the center target used during training. The decrease in performance while transferring an error classifier between similar yet different tasks requires further investigation because other works, on the contrary, achieved significantly similar good results while including new targets (e.g., Iturrate et al., 2015a, described in Section 6).

Also important to mention is that other works have not been so successful in detecting error severity. Iturrate et al. (2010b) have instructed four subjects to observe a 5-DOF robotic arm performing a reaching task to five color-coded areas (red—large error, green—target, and yellow—small error). They have explicitly told the subjects to evaluate the robot's motion based on the defined levels of severity. Even though error and correct trials could be differentiated, with an accuracy of 80%, they could not classify between small and large errors. Similarly, Spüler and Niethammer (2015) have attempted to identify different levels of execution error severity in a cursor control task by applying different angles (45°, 90°, or 180°). Their results did not confirm their hypothesis that a higher error amplitude would be observed for higher deflections. Authors have argued that using smaller angles (e.g., 15°, 30°, and 45°) could generate different results, especially if the degrees of errors are made very clear to the subject. But the previous study shows that explicitly instructing the subjects might still not be sufficient.

While also working with a cursor control task, but this time on a 2D setup, Iturrate et al. (2013a,b) have demonstrated the possibility of using ErrPs in an online system to learn to reach a goal location, either fixed or freely chosen by the subject, within a 5 × 5 grid from any starting position. In their approach, error-based learning consisted of two parts. First, the optimal policies for all possible targets in the grid were computed offline using Q-learning. Then, during online cursor control, the probability of having the goal location on each possible state was updated, based on EEG classification result, until a convergence criterion was satisfied.

Results of the reaching task show that the desired target could be reached within 25 and 21 steps for fixed and freely chosen goals, respectively. A random walker would require 150 steps in this grid. Moreover, there was a negative correlation between the time required to reach the target and the ErrP classifier accuracy. This result was expected and confirms that the effective use of ErrPs as reinforcement signals is highly dependent on their accurate classification. As the authors have mentioned, their approach exploits the structure of the optimal policies for each possible target location to compensate for the low ErrP detection rates and enable learning. They have also argued that their shared-control strategy could be applied to more complex scenarios. Because (i) it does not require extensive exploration of all possible trajectories and goals, and (ii) it does not impose extra workload on the user, who still only needs to monitor the system. On the other hand, a complete performance assessment of this approach requires a study with a representative number of subjects.

The proposed shared-control strategy has been extended to the continuous control of mobile robot navigation in a discretized 5 × 5 arena with continuous action and state-spaces (Iturrate et al., 2013b). A policy matching algorithm for inverse reinforcement learning to learn the desired target position used the error information to compute the likelihood^1^ values, differentiating rotation and linear actions. The error detection was based on a fixed high threshold value, penalizing targets in front or toward which the robot was turning when an error was detected. For the ErrP decoder calibration, they first collected trials with the subject pressing a button upon correct/wrong events (separately for each class). During the continuous evaluation of the system's performance, they have overcome the lack of a clear cue onset for the error detection with a classification based on overlapping windows of fixed length in an asynchronous fashion.

Only one subject participated in this preliminary experiment (no follow-up studies found) with freely chosen initial and target positions. Upon error detection, the navigation stopped for a second, and a new target was selected. Results over two runs show that the robot reached the goal positions within 60 and 121 s. Most erroneous events detection occurred during rotation motions, which allowed the robot to follow straight lines for most of its trajectory. Additionally, the proposed control strategy can recover from false positives and still reach the goal, as demonstrated along their second run (EEG data analysis has not been provided).

Focusing on neuroprosthetics control for real-life applications, Iturrate et al. (2015a) have evaluated three different BMIs under a new paradigm for learning the desired control command (i.e., motor behavior) to reach the target location. The motivation behind the proposal is to overcome the limitations imposed by systems that require learning to modulate specific brain signals (e.g., in motor imagery applications) to generate the corresponding motor commands for the prosthetic device. The tested BMI systems included a 1D cursor control with nine possible positions and targets on either extreme and a real and a simulated 2D robot control scenario with four possible target positions. The subject had to monitor each system acting to reach the defined goal position. Targets remained fixed within each run, and a new initial position was sampled every time the cursor reached the target. The detected ErrP was included in the reward function for an RL agent to learn the optimal control policies for reaching each of the predefined targets. The RL agent was based on iterative Q-learning starting with a random policy with updates upon EEG signal classification.

Data collected with twelve subjects show a difference waveform for the ErrP with larger positive and negative peaks at 300 and 500 ms at channel FCz, respectively. Interestingly, latency and magnitude differed significantly across experiments (cursor or robot), but classification accuracies were similar for all three (~73.5%). Online learning showed that, after four targets, the device achieved close to optimal, steady behavior and could reach the desired target from any starting position. On average, 12.38 ± 5.66, 12.46 ± 5.40, and 12.75 ± 6.63 targets were reached per run (a random walker would achieve 2.27 ± 1.56 and 2.32 ± 1.54, for cursor and robot scenarios, respectively).

The normalized number of actions required to reach the target converged to 1.19 ± 0.52 in the cursor experiment and 2.00 ± 0.76 and 1.97 ± 0.75 for the two-dimensional setups (optimal behavior would be 1). The system achieved above chancel level results after 10 and 15 actions, respectively, and the high correlation found between time and number of optimal learned actions suggested that performance improved continuously. Moreover, there was an increasing trend in the number of targets reached over time. The performance of experiments with the 2D environments that included two new target positions previously unavailable during ErrP calibration was significantly similar to the results for the practice targets. This result indicates that a re-training of the ErrP decoder is not required for unseen targets. Interestingly for the simulated robot, the number of optimal actions learned reached above chance level after only 4 actions while, in the real robot scenario, it took 14. Both paradigms had the same design. Why such a difference existed has not been further investigated.

Schiatti et al. (2018) have proposed to improve upon the approaches described so far and evaluated an online framework to learn both policy and target simultaneously as a robot performed a reaching task in a 2-D grid. The RL agent, implemented as a modified version of the Q-learning algorithm, directly received information about the target position and the output of the ErrP classifier as a reward to learn the optimal policy for each of the two possible targets. Afterward, the agent had to learn the intended target based on information on the routes and the decoded ErrPs. While Iturrate et al. (2015a) applied fixed values for the learning rate and the discount factor parameters, Schiatti et al. (2018) have applied different values in different learning phases.

To analyze the applicability of the proposed methods, the authors have performed offline simulations of both route and target learning for different ErrP detection accuracies (1.0, 0.8, and 0.6). Even for the lowest accuracy, including the ErrP information as reward proved more efficient than simple Q-learning. The ErrP-based reward formulation resulted in fewer steps necessary to reach the target during the first iteration (from 80 to 20) and generated routes of slightly smaller length at the beginning of the learning. During target learning, a similar learning behavior has been observed. Fewer steps were required to reach the current target (3−5, 7, and 15, for each simulated accuracy, respectively), and over 70% of the steps had a correctly identified target.

Eight subjects participated in online experiments, with half of them undertaking the experiment with only visual feedback and the other half receiving visual-tactile feedback. They have used different modalities to investigate their effect on ErrP decoding accuracy. The measured ErrP exhibited a significant negative peak around 270 ms, followed by another negativity around 650 ms after feedback onset for both feedback modalities. Other prominent peaks differed between the two conditions. The visual-tactile condition also usually generated larger peaks. However, ErrP classification accuracy did not vary significantly between feedback conditions (60 vs. 59%).

The learning performance also did not present any significant differences based on the type of feedback used. However, for the visual-tactile modality, corrections did happen in earlier steps during route learning (5–8th vs. 8–11th iteration). During target learning, the authors have reported a more uniform average number of correct steps (even though it ranges from 60–80%). Generally, learning performance seems coherent with the simulated results for a 60% error detection. One contribution of this work, from our perspective, is the simulation performed. We believe that performing such simulations for the learning approaches will help us establish boundaries for error-based learning/adaptation in different applications. Moreover, it could facilitate the comparison of approaches.

Learning to identify the desired target and the optimal policy to reach it was analyzed in the works so far revised in this section. Unlike (Chavarriaga et al., 2007; Chavarriaga and Millan, 2010) and Iturrate et al. (2015b) (listed in Section 5) that applied an offline likelihood-based framework for learning the optimal policy for reaching the desired target in the 1D cursor control, the works described in this section have used an RL-based framework for both offline (Iturrate et al., 2013a,b) and online (Iturrate et al., 2015a) policy learning as well as for online target learning. In the robot control setup, Iturrate et al. (2013b, 2015a) and Schiatti et al. (2018) have expanded the setup used previously (Iturrate et al., 2010a to a 2D configuration. Tables 2, 3 summarize the problem definition in each work.

In recent work, Akinola et al. (2020) have indirectly applied the ErrPs to accelerate robot skill learning in an RL setup with sparse rewards. First, the ErrP information drove the online training of a human feedback policy. A neural network trained using supervised learning to map state-action pairs to the human label given by the ErrP classifier, and that learned the probability of positively rewarding each action. In a second step, this policy guided exploration at the beginning of learning. As time proceeded and the actual RL policy became able to learn despite the sparse rewards, the human policy application stopped, and policy learning continued in an on-policy fashion, using the PPO algorithm (Schulman et al., 2017). The motivation for their approach was that, while previous works have successfully demonstrated the viability of using brain signals as feedback for learning, none of the systems achieved an autonomous operation with the same performance once the human feedback was no longer available.

Akinola et al. (2020) have performed experiments using simulated robot navigation in an environment with obstacles and a fixed target position. They have modeled the RL problem with a discrete action space to facilitate the subject's assessment and defined three actions: moving forward and turning (left or right). The state-space considered remained continuous and was given in terms of laser range measurements, displacement to the goal in polar coordinates, and the yaw angle of the robot. The sparse reward function for this navigation problem specified punishments for each action not leading to the target reached and collisions and a reward for reaching the target. They have additionally considered an informative reward function extending the sparse formulation with the euclidean distance from the goal and the orientations.

The learning algorithm performance has been tested under two experimental conditions with a fixed and a variable starting position. Simulation results show better learning performance for higher ErrP accuracies (0.7, 0.6, 0.55) in the proposed algorithm extending the sparse reward with the human policy, in the beginning, to guide exploration toward the goal. Either with a fixed or variable starting position, learning based only on the sparse reward function presented worse performance [given in terms of success weighted by normalized inverse path length (SPL)]. An expected result, since the agent only rarely stumbled on the target, receiving a positive reward that would be informative enough to learn the given task. The complex reward function also guided task learning, as predicted, but not significantly better than the proposed algorithm, which has the advantage of not requiring expert knowledge.

Seven subjects participated in an online experiment under the variable start condition. Results are reported for five participants only, for whom the ErrP decoding accuracy was high enough to guide policy learning. Recorded neural data analysis has not been provided. Results are similar to the simulations, showing comparable performance between the proposed method and the richer reward function. Nevertheless, the authors show that five runs with the proposed method achieved less variation.

Limitations of the approach are the discretized action space and the single target. Future work should focus on approximating realistic applications. It would also be interesting to further analyze how the ErrP decoding accuracies affected online learning. Finally, one could examine the usability and advantages of training a human feedback policy and consider, for example, the relevance of such an approach for BMIs.

The same approach of using the error signal to train a reward predicting function to replace the human observer has been tested by Luo et al. (2018), who used it to guide learning in four difficult RL environments that are well-known in robotics applications: Cheetah, Reacher, Hopper, and Ant (Christiano et al., 2017). The environment specifications (state/action spaces and reward functions) have not been reported. Twelve subjects took part in the experiments, three per environment. With 70% error detection accuracy, learning based on the rewards provided by the error-based reward function trained did not achieve good performance, reaching lower reward values than the ones obtained by what the authors called human and synthetic queries for all subjects and environments. The authors have claimed that collecting more trials improved the results. However, it is unclear how they have performed the comparisons.

### 6.2. Learning While Interacting

All works mentioned in the previous section have used observation ErrPs to improve brain-machine interface performance. However, it is also relevant to study whether such a reinforcement learning framework can be applied when the subject actively interacts with the system. Such conditions could represent a more realistic application scenario for BMIs, especially when dealing with patients. The works summarized in this section have analyzed such conditions.

Roset et al. (2014) have proposed an adaptive BMI as a proof-of-concept system for augmented rehabilitation involving hand grasp/open movements. The system implements an actor-critic architecture wherein the actor decodes the motor potentials to determine the desired hand movement, and the critic relies on its ability to detect ErrPs to provide feedback to the actor. The ErrP decoder was trained using supervised learning with an error rate of 50%, and the weights for the actor's neural network were randomly initialized in the first closed-loop session, updated after each trial, and used as initial values for the subsequent session.

Results over four sessions on different days show that the actor's cumulative classification accuracy increased over time from chance level and approached the classification accuracy of the critic (68.8% and 64.2%, for control and SCI subjects). This successive improvement over sessions suggests that the subjects resumed their rehabilitation progress. The authors report that the updates on the actor's neural network weights reduced after the second session and continued throughout the trials, suggesting that performance would not worsen over time and instead could further improve. For comparison, with static weights trained using data from the previous session, the accuracy is initially above the chance level. But it decreases over time to below 60%.

The measured ErrP at the Cz electrode was consistent with results reported in other interaction tasks. Unlike other studies, this setup has also been tested in a patient with spinal cord injury (SCI). Though significantly above the chance level, the performance was slightly worse when compared to the control subject, which could be explained by the lower ErrP decoding accuracy.

The proposed setup seems interesting for everyday use of rehabilitation systems since it does not require daily initialization and seems able to improve over time. To fully address its advantages, it is necessary to expand this setup to a study with a representative number of subjects and include quantitative analyses of the adaptation observed.

Kim et al. (2017) have also focused on more realistic applications in which the subject not only observes the system but actively interacts with it. They have implemented a human-robot interface that allowed subjects to freely choose between three hand gestures to control a robotic arm. The robot had no prior information about the meaning of each gesture and learned it based on gestures features extracted from a Leap Motion Controller sensor (LeapMotion, 2021). In other words, the system simultaneously learned how to recognize human gestures and the correct gesture-action mapping (compare with Roset et al., 2014).

As a learning algorithm, the proposed system implements a contextual bandit approach (Li et al., 2010), which considers the context provided by the gestures to choose the appropriate action at each interaction trial. The action selection strategy improves with time, based on the information from the ErrP classifier. To further improve learning robustness, positive feedback received higher weights by using a data augmentation approach in which the ErrP classifier made two decisions for the same trial. With the application of two overlapping windows around the same feedback event, the detection of positive events became more reliable since only when the classification for both windows was correct positive feedback was generated. This design also helped overcome the low number of erroneous events to train the ErrP classifier. Additionally, they speeded up calibration with a classifier transfer approach from observation to interaction ErrPs. Performance slightly decreased but achieved a high-enough accuracy during online experiments to moderate effective learning. To reduce the number of errors at the beginning of the learning, the RL agent was pre-trained, for each subject, using up to three gesture-actions pairs.

Experiments were performed with seven (nine) subjects with simulated and robot scenarios. EEG data analysis revealed ErrPs with positive and negative peaks at fronto-central electrode FCz with slightly delayed latencies of 332 and 540 ms (real robot) and 504 and 584 ms (simulated robot). Between scenarios, the peaks amplitudes did not differ significantly. But the positive peak latency was significantly larger, and the negative peak was also significantly enhanced and had lower latency for the simulated robot scenario. The authors have attributed the delayed latencies and different results to the action execution speed, which was much faster with the simulated robot. Since the error detection used the action onset, subjects might have only detected it at the end of the action execution. Still, online balanced ErrP classification accuracies reached ~90% over all subjects for both scenarios. Further analysis of the robot behavior learning progress showed that false negatives on ErrP detection had a higher impact on learning than false positives. This observation is a direct consequence of the proposed framework favoring true positives.

Learning performance in terms of the number of accumulated errors made throughout the experiment showed similar results for simulated and real robot scenarios (9.57 ± 0.32 over 90 actions vs. 4.86 ± 1.21 over 60 actions). For the simulated robot, two subjects experienced a higher number of errors and, consequently, a slow stabilization during online learning. However, accumulated errors were fewer for the second part of the experiments. Statistical tests showed significant differences in the total number of errors between the first and second half, meaning that the agent committed fewer errors as learning proceeded, and it reliably mapped gestures into correct actions according to the subject's assessment. For the real robot, the same behavior has been observed but only descriptive results are provided because of the reduced number of trials in the second phase.

According to the authors, the proposed learning framework has the advantage of not being limited by the number of possible actions. Nonetheless, further studies are required to evaluate learning convergence when too many options (i.e., a large action space) are available. Moreover, one could consider applications involving asynchronous ErrP detection and continuous control. As the late ErrPs components indicate, it is hard to precisely know when the subject realized the error after the robot started an action. Therefore, reliably detecting such signals in asynchronous setups could bring the approach closer to more complex and realistic interaction scenarios, in which a clear event onset might not be possible.

Another interesting aspect of this proposal is the possibility of re-learning if the subject changes the gesture meaning during learning. The authors have not tested this concept, but they have investigated the possibility of incorporating new gestures in a follow-up work (Kim et al., 2020). In this case, the authors have only focused on the real robot application with essentially the same experimental setup. They have analyzed the effect of adding a new gesture on online learning by instructing the subjects to perform only three gestures, and after an acoustic signal, add the new one. Subjects performed experiments under two conditions: with a pre-trained model (warm-start) and without pre-training (cold-start) (please note that results were reported based on different total number of trials: 90 and 120, respectively).

Comparisons showed that pre-training the model with one or two gesture-action pairs significantly improved online learning (~30 vs. ~10% mapping errors over all trials for no- vs. pre-training conditions. The values were approximated from the provided plot). The accumulated errors reduced over time, even though a slight increase was observed immediately after adding the new gesture. On the other hand, with the untrained model, the number of errors was high in the first third of the experiment, and significantly reduced with time, even after including the new gesture. This result suggests that collecting more information about the three initial gestures somehow compensated for the lack of pre-training and contributed to accumulated error reduction. In line with this observation, the slight performance decrease in the last part of the experiment also led the authors to conclude that increasing learning accuracy after inserting the new gesture would require more trials. Finally, the authors have also investigated the correlation between ErrP-classification performance and learning performance. It is not surprising that they have found a high correlation and that the cold-start model was more sensitive to error detection performance. An analysis of the effect of poor gesture recognition remains an open question.

In another work implementing an error-based learning scenario while subjects interact with the environment, Ehrlich and Cheng (2018) have applied the ErrPs as mediating signal for human-robot co-adaptation. While the subject adapted his actions by reflecting the robot's behavior, the robot itself also adapted its actions based on the subject's choices.

Their experimental setup consisted of a guessing game involving objects positioned between subject and robot. The robot chose one object and gazed at all three plus the subject, who had to observe the pattern and guess which object was the robot's choice, without hints of what to look for. The proposed idea was to show that a robot-human co-adaptation can be mediated by the ErrP generated when the subject wrongly guesses which object was chosen by the robot. Based on this negative feedback, the robot should adapt online its gazing policy to enable correct guessing from the human in the future, as both try to achieve a consensus.

Policy adaptation used policy gradient methods and was mediated by the error information. This way, to compute the next policy, the algorithm combined the parameters of the current policy with the weighted empirical distribution information of the current trial. They expected that the more common state-action pairs would be more relevant for correct or wrong subject guesses. Hence, with this formulation, information about the gazing policy the subject has observed could be considered while updating the policy.

Thirteen participants participated in the experiments. The measured ErrP showed an N2-P3 complex with a fronto-central distribution and could be classified online with an average accuracy of 81.8%, which sufficed to drive co-adaptation. Successful adaptation reported in terms of the guessing accuracy during each adaptation run shows that it increased over time from the chance level to up to 70–90% within 10–40 trials. Moreover, the number of gazing actions the robot took before the subject informed the guess decreased 15–27%. This reduction indicates that adaptation of robot's behavior based on the subject's information brings along a more efficient human-robot interaction, as subjects could respond faster and more accurately. Analysis of policy convergence in terms of the difference between subsequent policy iterations was in line with the previous results and showed a decreasing trend, indicating convergence over time.

A comparison of the learned policies with and without explicit human feedback *via* keypress provided no indications that this feedback is required. However, given the limited data for successful adaptation in this single run, the authors have not reported further analysis from this data. They have also analyzed the generated policies and identified two types: what they defined as fixation and nodding behavior. Following the fixation policy, the robot tended to focus more often on the desired object. On the other hand, the nodding policy made it gaze more often between the human and the selected object only. The convergence to two policies seems to be a direct consequence of the experimental setup used, which did not expect a specific behavior from the robot or the human. The authors hypothesize that future studies that analyze why only two strategies appeared could provide insights into how we process information and learn.

Similar to the works that used the ErrPs to guide robot skills learning, the results show that a significant positive correlation exists between co-adaptation performance and ErrP decoding accuracy. Interestingly, however, some cases of unsuccessful co-adaptation have been observed even with a high (> 75%) ErrP decoding accuracy. According to the authors, it could be related to subject attention and motivation or the setup itself. As they have explained, the proposed strategy focused on quick learning convergence, but not necessarily to a global optimum. It provided flexibility to the setup, allowing learning to be somehow robust against chances on the subject's strategy on the run. On the other hand, authors hypothesized that this might have caused instabilities and even promoted quick unlearning. They suggest using an adaptive learning rate based on ErrP detection, under consideration that such control of the learning process is recommendable. A deeper investigation should also be performed considering longer co-adaptations runs than the 50 iterations considered in this study. Authors claim that, because of the gradient-based method for policy learning, the proposed setup should generalize and scale to more complex HRI scenarios.

## 7. Discussion

We presented a review of studies that utilize the detection and classification of error-related potentials in a reinforcement learning framework to go beyond error correction and use these signals directly for learning. These works report improved BMIs' performance and demonstrate that the RL framework is capable of learning and adapting related mappings based on error-related potential in a self-organized manner. Nevertheless, there is still room for improvement, and future research should address specific issues.

As expected and reported by many of these works, the effective use of ErrPs as feedback signal highly depends on their reliable detection on a single-trial basis. Therefore, many experimental protocols usually start with a calibration phase to collect trials and train a subject-specific ErrP decoder under very controlled error rates (usually around 20–30%). Calibration is a time-consuming task that usually takes between 20 and 30 min since a considerable number of trials for each class (error and correct) have to be collected. Also critical is that error trials usually happen less often than correct ones, resulting in an imbalanced training set and a class bias in the error classifier.

Many works have proposed a calibration-free setup by applying classifier transfer between subjects. Schonleitner et al. (2019, 2020) have shown that the generalized model achieved acceptable performance, and further using supervised or unsupervised adaptation strategies could additionally boost performance. Lopes-Dias et al. (2020) have proposed a generic classifier that achieved comparable performance to the subject-specific model, and Lopes-Dias et al. (2021) have transferred a generic classifier between healthy subjects and subjects with spinal cord injury. But, even though their model achieved acceptable performances in all these setups, performance still decreased after transfer. Kim et al. (2017) have shown that it is also possible to transfer classifiers between different error types. The advantage, in this case, is that using an observation error for calibration enables collecting more trials since less time per trial is required than when the subject is also interacting with the system.

More recent works have also focused on the application of convolutional neural networks as an alternative to the widely used Support Vector Machine or Linear Discriminant Analysis models (Behncke et al., 2018; Swamy Bellary and Conrad, 2019; Gao et al., 2020). Results show a slightly increased accuracy, which is again a strong indicator that it is not the machine learning techniques that determine the limits of accuracy in the error-related potentials classification problem. The relation between the provoking event and the related measured signal (occurrence, amount, time course, etc.) in the brain has to be better understood and precisely determined.

The error rate used during the calibration phase also seems to affect error amplitudes and, as a consequence, the decoding, as shown by Chavarriaga and Millan (2010), who have compared 20% and 40% error rates, with less prominent peaks for the second condition. On the other hand, Pezzetta et al. (2018) have shown that ErrPs are also generated when the error rate is higher (80%), but they have not reported classification comparisons. Therefore, future systems design should also analyze which other interface factors might affect error-based learning.

However, an interesting question is how accurately does the error classifier actually have to be? Sutton and Barto (2018) have shown that reinforcement learning algorithms can learn optimal policies even with reward uncertainties. Chavarriaga et al. (2014) and Iturrate et al. (2015a) also claim that the ErrP decoding on a single-trial basis does not have to be perfect. According to them, an above chance level accuracy is enough to teach the system the correct motor behavior, provided that the initial system performance is already acceptable to the subject. Considering this aspect can model the efforts to increase ErrPs classification accuracy.

On the other hand, one might also consider addressing the uncertainties around the error generation in the brain itself and how they propagate through the learning pipeline. Error-related signals should be generated in the brain even when the subject is not actually conscious of them (Nieuwenhuis et al., 2001). However, what if it is also possible that, on a single-trial basis, a distinguishable error signal is, in fact, not generated at all? If this would be the case, improvements on BMI interfaces would already be fundamentally limited. In terms of learning, considering such uncertainties might help develop more robust systems. In this work, we have only focused on the existing non-invasive-based literature. Exploring available data and findings provided by studies using invasive techniques might also contribute to better establishing the boundary conditions for the reliable detection of such signals on the scalp. Moreover, especially since the applications focusing on using such signals rely on their single-trial detection, it could be interesting to start looking at the single-trial characteristics of the ErrPs. We believe that understanding the conditions under which such signals are generated can also guide the specification of a more suitable preprocessing pipeline and an accurate classifier.

In the studies reviewed, the measured ErrP has been commonly reported by means of the grand average ERP as it is part of the fundamentals of the ERP theory. Still, there is no standard in the studies for reporting the averages. The difference grand average (error minus correct) over all trials and subjects is widely used and provides information about the shape of the elicited ErrP. It has, however, the limitation of masking the inter-trial and subject variabilities, which are particularly pertinent since the applications require the single-trial detection of such signals. On the other hand, only a few studies have provided an additional statistical comparison for the significance of the observed differences. The grand averages for the error and correct trials have also not always been reported. Additional ERP analyses such as topographical scalp distribution and source localization have in some cases been covered. Such analyses could help the further characterization of the ErrP and provide additional means for comparison across experiments. None of the studies considered have attempted to analyze the properties of the respective ErrPs in the frequency domain to characterize the frequency range modulations. This information could also be used, e.g., to tailor frequency-domain-based classifiers.

Moreover, as it can also be seen in the works listed here, the errors waveshape exhibit differences in terms of components observed, as well as their respective latencies, even when using very similar tasks. How well these waveforms generalize across subjects, tasks, experimental protocols, and feedback modalities have to be better understood. Aspects such as subject attention, engagement, motivation, and fatigue have to be systematically investigated under the described protocols to quantify how they affect the modulation of the ErrPs and influence decoding and learning performance.

The ErrP-based BMI systems summarized here have consistently reported that the ERP components associated with error processing and performance monitoring generally show a fronto-central distribution, with the anterior cingulate cortex (ACC) as their most likely source. Future BMI research should also focus on the underlying cognitive processes that generated the measured components. Analyzing the connection between measured ERP components and corresponding cognitive sources can not only improve our current understanding of the neural correlates of performance monitoring, error processing, and learning in the brain but also contribute to the development of robust BMI systems that intend to use such signals.

Evaluation metrics for the ErrP classification performance also vary among the studies reviewed. We also believe that using only the overall classification accuracy as metrics is not suitable considering that the ErrP classification constitutes a highly imbalanced binary classification problem. At least the true-positive and negative rates should also be provided, and other metrics such as the balanced accuracy, the F1 score, or the precision-recall AUC score could also be considered.

Apart from the consistently reported correlation between the error classification accuracy and the learning performance over the summarized works, another common aspect is that the adopted experimental protocols considered error-related potentials time-locked to discrete feedback events. As a consequence of this choice, the experimental paradigms used were also limited to discrete tasks and action spaces.

Most of the works have chosen a simple 1D or 2D reaching task of similar dimensions as the target application. However, even though the task setups were quite similar, comparing their results is somehow limited because they have focused on trying different modifications of these simple setups under different conditions. This emphasizes how research on the use of error potentials as reinforcement learning signals is still in its initial phase and has so far mostly focused on demonstrating the feasibility of using ErrPs to learn a simple, well-defined task within a very controlled scenario. Hence, future work should further explore each different possibility and provide comparable frameworks.

As for the discrete feedback limitation, future work should also attempt to expand these paradigms to continuous setups. Different works have already shown, for example, that ErrPs can also be detected under continuous feedback in combination with discretized events for error decoding (Spüler et al., 2015; Kreilinger et al., 2016). Alternatively, one could also consider asynchronously detecting such signals. This approach does not require a defined time-locking event and can expand error-based BMIs to more complex scenarios. Omedes et al. (2015a) have shown, for example, that errors in the start or middle of a target reaching trajectory could reliably be detected asynchronously when the subject observed the cursor on the screen. Later, authors compared error signals after sudden and gradual changes in the cursor trajectory but were not able to distinguish between both types of errors as no discernible error pattern could be identified for the gradual changes (Omedes et al., 2015b). However, they have shown that it is possible to train an asynchronous detector for sudden errors and use it to detect gradual errors.

More recently, Lopes Dias et al. (2018) have used a continuous cursor control task with a joystick to analyze the error signals generated under two continuous feedback conditions: a normal and a jittered. Both errors could be classified against the no error condition, but it was not possible to distinguish between them. In any case, asynchronous detection of error events (including both feedback conditions) was still possible. These results suggest that their approach could be reliable, for example, if brain signals are directly used to control the devices. The same authors have demonstrated that online asynchronous detection of execution ErrPs during interaction with a real robot is possible, not only for healthy (Lopes-Dias et al., 2019) subjects but also for spinal cord injured patients (Lopes-Dias et al., 2021). This observation shows the feasibility of also using such systems in more realistic applications.

In summary, this paper reviews the current studies applying error-related potentials to improve brain-machine interface performance. Most specifically, we have focused on the use of such brain signals in reinforcement learning frameworks. Starting from a top-down perspective, we have systematically discussed different types of ErrPs, how to detect them, and considered all relevant contributions within the outlined focus, describing strengths and weaknesses and assessing the limitations of the approaches. Furthermore, we tried to answer the question of which accuracy is sufficient to trigger a learning process utilizing ErrPs. However, it becomes clear that the field is still dealing with very fundamental open questions that make a rigorous comparison between the different studies quite difficult. It is still unclear whether a significant and prominent ErrP occurs every time, what is the ratio between occurrence and detection and the resulting maximum bound for classification, and what accuracy limit is needed to successfully drive the RL process. We have attempted to highlight these open questions and, to the best of our knowledge, describe the current state of the research.

The studies evaluated have demonstrated that ErrPs provide relevant information that can be used to guide agent behavior learning. Nonetheless, future work is necessary regarding higher error decoding accuracies to increase the reliability of the feedback signal. A comprehensive performance assessment of the proposed approaches additionally requires studies with a representative number of subjects and the establishment of comparable and meaningful performance measures to support the evidence of learning/adaptation under these paradigms. Although the studies used suitable metrics to evaluate the respective learning processes, which are sufficient to measure performance, the problem of not knowing precisely the relationship between occurrence and detection of ErrPs remains and provides an inherent uncertainty with respect to the evaluation of the overall RL process. It would be helpful, e.g., to systematically evaluate the learning process with simulated ErrPs. This would not only provide a relative measure for the learning process but also make it possible to calculate the minimum threshold for the occurrence-to-detection ratio needed to learn. Experimental setups should also be extended to larger state/action spaces and continuous feedback scenarios with asynchronous detection.

Last but not least, BMI systems were originally thought for subjects with different disabilities, and are still mainly focused on them. Hence, future work should consider extending error-based reinforcement learning frameworks to applications focused on this target group.

## Author Contributions

AXF and II defined the paper scope. AXF, CK, and II revised the manuscript. All authors contributed to the article and approved the submitted version.

## Funding

This work was supported by the Ministry of Economics, Innovation, Digitization and Energy of the State of North Rhine-Westphalia and the European Union, grants GE-2-2-023A (REXO) and IT-2-2-023 (VAFES).

## Conflict of Interest

The authors declare that the research was conducted in the absence of any commercial or financial relationships that could be construed as a potential conflict of interest.

## Publisher's Note

All claims expressed in this article are solely those of the authors and do not necessarily represent those of their affiliated organizations, or those of the publisher, the editors and the reviewers. Any product that may be evaluated in this article, or claim that may be made by its manufacturer, is not guaranteed or endorsed by the publisher.

## Footnotes

^1^The learning framework used is described in Figure 4. This work is a follow-up, and to connect it to its previous work, we have chosen to keep it in this section.
