# Supplementary material for: Error-Related Potentials in Reinforcement Learning-Based Brain-Machine Interfaces
Source: Front Hum Neurosci. 2022 Jun 24;16:806517. doi: 10.3389/fnhum.2022.806517 (PMC9263570; doi:10.3389/fnhum.2022.806517)
Supplement: Supplementary file 1 [file Data_Sheet_1.pdf]

# Supplementary Material

**Table S1.** Overview of brain-machine interface systems that use error-related potential for error correction. This table summarizes works that have applied the error-related potential information to propose an error correction to improve BMI performance. Please notice that the "Errp type" column represents our own classification of the error type used, which we defined considering the experimental setup, if authors did not explicitly mention the error type being considered.

| Application | Ref. | #subjects | User task | ErrP Application | ErrP type (possibly) |
|---|---|---|---|---|---|
| cursor control | Ferrez and Millán (2008) | 2 healthy | cursor control using MI | used to stop movement if cursor movement was wrong | interaction ErrP |
| | Mousavi et al. (2017) | 10 healthy | cursor control using MI | ErrP used in MI classifier to better detect whether cursor is moving towards or away from the target | interaction ErrP |
| | Kreilinger et al. (2016) | 10 healthy (offline) 4 healthy (online) | cursor control using MI | used to discard MI trial, which had to be repeated by user | interaction ErrP |
| BCI Spellers | Schmidt et al. (2012) | 12 healthy | focus on intended character | ErrP used to delete the wrong character and restart trial | interaction ErrP |
| | Chavarriaga et al. (2016) | 4 healthy | monitor cursor movement | ErrP used to indicate that cursor did not move towards the intended character | interaction ErrP |
| | Dal Seno et al. (2010) | 3 healthy | focus on intended character | ErrP used to cancel letter selection | interaction ErrP |
| | Margaux et al. (2012) | 16 healthy | focus on intended character | ErrP used to delete the wrong character. In the automatic correction approach, the second best guess of the classifier was used | interaction ErrP |
| | Cruz et al. (2018) | 9 healthy 1 tetraplegic | focus on intended character | Error used to delete wrong character and replace it by second highest target score but if this second feedback elicited an ErrP again, the first spelled symbol was re-selected | interaction ErrP |
| robot control | Bhattacharyya et al. (2014) | 5 healthy | control a robotic arm | ErrP used as feedback response to undo action if motor imagery was classified wrongly (robot moved to wrong direction) or to compensate for offset error while reaching the arm end position | interaction ErrP |
| | Bhattacharyya et al. (2017) | 12 healthy | control a robotic arm | ErrP used align robotic link by turning/moving the link in reverse direction by an experimentally determined fixed angle/distance. | interaction ErrP |
| | Rakshit et al. (2016) | 5 healthy | control a robotic arm | ErrP used to align arm with target position in case of misalignment | interaction ErrP |
| | Penaloza et al. (2014) | 5 healthy | observe robot actions through camera | ErrP used to select second-best action if first one elicits an ERN signal | observation ErrP |
| | Salazar-Gomez et al. (2017) | 12 healthy | observe robot | ErrP used to correct erroneous actions of a robot | observation ErrP |
| | Chavarriaga et al. (2010) | 7 healthy | make gesture and observe computer response | ErrP used in re-calibration (just detection and theoretical improvement analysis) | interaction ErrP |
| | Putze et al. (2015) | 20 healthy | make gesture and observe computer response | Evaluate different strategies to use ErrP to correct the gesture recognition | interaction ErrP |
| | Lopes-Dias et al. (2019) | 15 healthy | control robotic arm towards a target with right hand movement | ErrP used to undo offset in robot position | execution ErrP |
| | Förster et al. (2010) | 7 healthy | make gesture and observe computer response | ErrP used in re-calibration (just detection and theoretical improvement analysis) | interaction ErrP |
| prosthetic and exoskeletons | Zhang et al. (2018) | 8 healthy | control lower-limb exoskeleton | ErrP used as feedback to confirm the command sent | interaction ErrP |
| | Rotermund et al. (2006) | N/A | indent to a movement to occur and observe the movement of the prosthetic arm | ErrP used to adapt the decoding of the intended arm movement (ErrP is the hypothetical evaluation signal) | N/A |
| | Perrin et al. (2010) | N/A | monitor wheelchair positions | ErrP used to re-prompt command until user accepted the suggestion | observation ErrP |
| miscellaneous | Yousefi et al. (2019) | 10 healthy | perform cognitive tasks | ErrP used to correct classification output displayed to user | interaction ErrP |
| | Parra et al. (2003) | 7 healthy | discriminate between two visual stimuli by pressing one of two buttons | ErrP used to correct erroneous response of subjects | response ErrP |

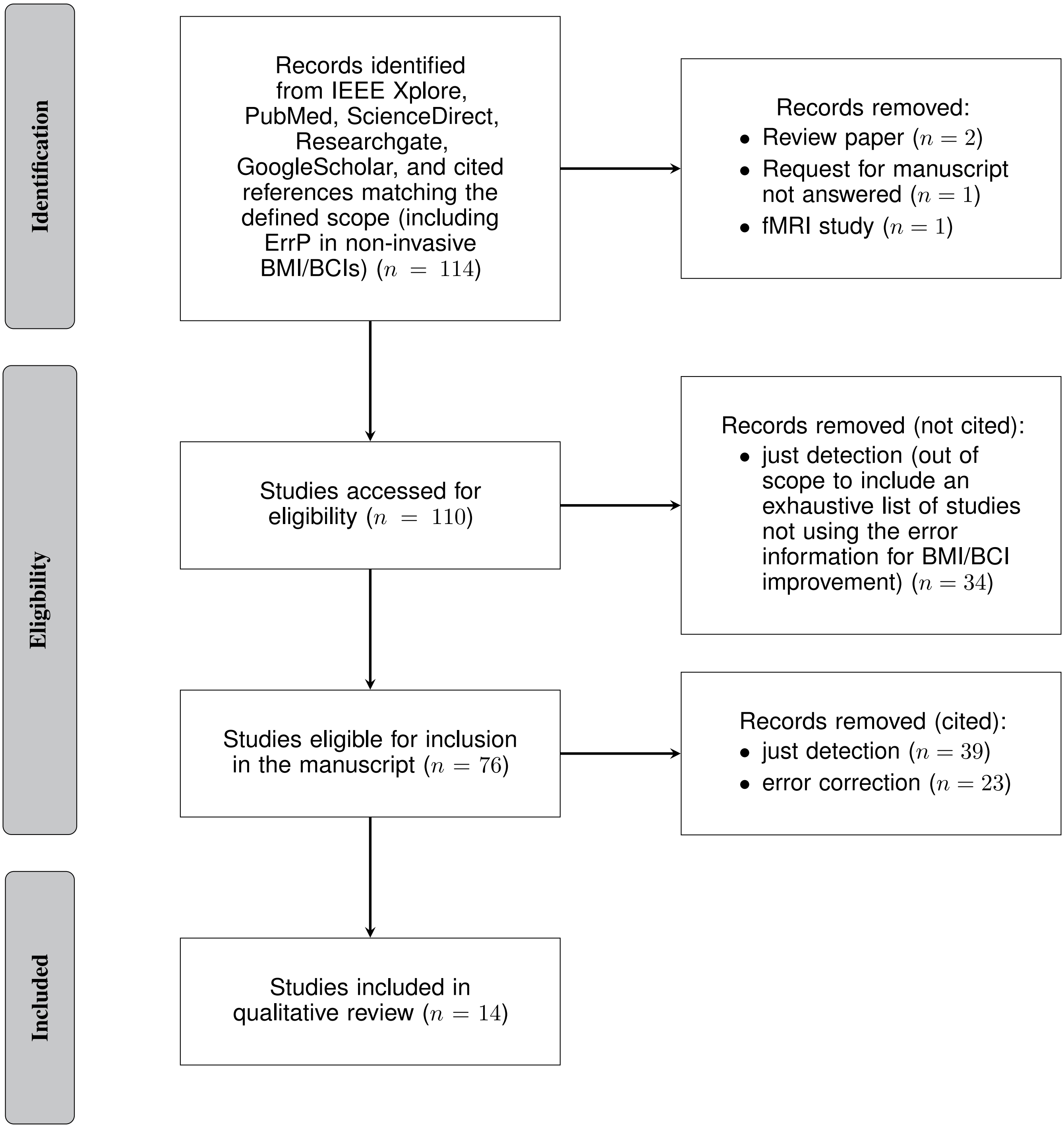


Figure S1: Flow diagram (based on the flow diagram from the PRISMA statementPage et al. (2021)) for the review process applied in this work. We included the relevant literature within the scope specified for this review (i.e., ErrP, EEG, BMI, BCI, RL), considering different databases and search engines, including IEEE Xplore, PubMed, ScienceDirect, Researchgate, GoogleScholar, and cited references. GoogleScholar was also used to identify papers citing the works already found. Among the entries found, 110 studies were screened. From each work, a group of predefined information was extracted from the manuscripts. General information such as #subjects, EEG signals (e.g., ErrP, or P300), subject's task (e.g., observe robotic arm), application (e.g., cursor, robot control) was considered. The ErrP application (e.g., just detection, error used to stop cursor movement, to delete, or as a reward in RL, etc.) was carefully extracted by a researcher considering how exactly according to the authors the ErrP has been incorporated in their setup. Since it is out of the scope of this manuscript to include an exhaustive list of all studies not applying the detected ErrP for BMI learning (or improvement), we have only included some of these works throughout the manuscript, prioritizing more recent works and applications that had not yet been mentioned.

## REFERENCES


Bhattacharyya, S., Konar, A., and Tibarewala, D. N. (2014). Motor imagery, P300 and error-related EEG-based robot arm movement control for rehabilitation purpose. *Medical & Biological Engineering & Computing* 52, 1007–1017. doi:10.1007/s11517-014-1204-4

Bhattacharyya, S., Konar, A., and Tibarewala, D. N. (2017). Motor imagery and error related potential induced position control of a robotic arm. *IEEE/CAA Journal of Automatica Sinica* 4, 639–650. doi:10.1109/JAS.2017.7510616

Chavarriaga, R., Biasiucci, A., Forster, K., Roggen, D., Troster, G., and Millan, J. d. R. (2010). Adaptation of hybrid human-computer interaction systems using EEG error-related potentials. In *2010 Annual International Conference of the IEEE Engineering in Medicine and Biology* (Buenos Aires: IEEE), 4226–4229. doi:10.1109/IEMBS.2010.5627376

Chavarriaga, R., Iturrate, I., and Millan, J. D. R. (2016). Robust, accurate spelling based on error-related potentials. *Proceedings of the 6th International Brain-Computer Interface Meeting* doi:10.3217/978-3-85125-467-9-15. ISBN: 9783851254679 Publisher: Verlag der Technischen Universität Graz

Cruz, A., Pires, G., and Nunes, U. J. (2018). Double ErrP Detection for Automatic Error Correction in an ERP-Based BCI Speller. *IEEE Transactions on Neural Systems and Rehabilitation Engineering* 26, 26–36. doi:10.1109/TNSRE.2017.2755018

Dal Seno, B., Matteucci, M., and Mainardi, L. (2010). Online Detection of P300 and Error Potentials in a BCI Speller. *Computational Intelligence and Neuroscience* 2010, 1–5. doi:10.1155/2010/307254

Ferrez, P. W. and Millán, J. d. R. (2008). Simultaneous Real-Time Detection of Motor Imagery and Error-Related Potentials for Improved BCI Accuracy. *Proceedings of the 4th international brain-computer interface workshop and training course* , 7

Förster, K., Biasiucci, A., Chavarriaga, R., del R. Millán, J., Roggen, D., and Tröster, G. (2010). On the Use of Brain Decoded Signals for Online User Adaptive Gesture Recognition Systems. In *Pervasive Computing* (Berlin, Heidelberg: Springer Berlin Heidelberg), vol. 6030. 427–444. doi:10.1007/978-3-642-12654-3_25. Series Title: Lecture Notes in Computer Science

Kreilinger, A., Hiebel, H., and Muller-Putz, G. R. (2016). Single Versus Multiple Events Error Potential Detection in a BCI-Controlled Car Game With Continuous and Discrete Feedback. *IEEE Transactions on Biomedical Engineering* 63, 519–529. doi:10.1109/TBME.2015.2465866

Lopes-Dias, C., Sburlea, A. I., and Müller-Putz, G. R. (2019). Online asynchronous decoding of error-related potentials during the continuous control of a robot. *Scientific Reports* 9, 17596. doi:10.1038/s41598-019-54109-x

Margaux, P., Emmanuel, M., Sébastien, D., Olivier, B., and Jérémie, M. (2012). Objective and Subjective Evaluation of Online Error Correction during P300-Based Spelling. *Advances in Human-Computer Interaction* 2012, 1–13. doi:10.1155/2012/578295

Mousavi, M., Koerner, A. S., Zhang, Q., Noh, E., and de Sa, V. R. (2017). Improving motor imagery BCI with user response to feedback. *Brain-Computer Interfaces* 4, 74–86. doi:10.1080/2326263X.2017.1303253

Page, M. J., Moher, D., Bossuyt, P. M., Boutron, I., Hoffmann, T. C., Mulrow, C. D., et al. (2021). Prisma 2020 explanation and elaboration: updated guidance and exemplars for reporting systematic reviews. *Bmj* 372

Parra, L., Spence, C., Gerson, A., and Sajda, P. (2003). Response error correction-a demonstration of improved human-machine performance using real-time EEG monitoring. *IEEE Transactions on Neural Systems and Rehabilitation Engineering* 11, 173–177. doi:10.1109/TNSRE.2003.814446

Penaloza, C. I., Mae, Y., Kojima, M., and Arai, T. (2014). BMI-based framework for teaching and evaluating robot skills. In *2014 IEEE International Conference on Robotics and Automation (ICRA)* (Hong Kong, China: IEEE), 6040–6046. doi:10.1109/ICRA.2014.6907749

Perrin, X., Chavarriaga, R., Colas, F., Siegwart, R., and Millán, J. d. R. (2010). Brain-coupled interaction for semi-autonomous navigation of an assistive robot. *Robotics and Autonomous Systems* 58, 1246–1255. doi:10.1016/j.robot.2010.05.010

Putze, F., Amma, C., and Schultz, T. (2015). Design and Evaluation of a Self-Correcting Gesture Interface based on Error Potentials from EEG. In *Proceedings of the 33rd Annual ACM Conference on Human Factors in Computing Systems* (Seoul Republic of Korea: ACM), 3375–3384. doi:10.1145/2702123.2702184

Rakshit, A., Lahiri, R., Ghosal, S., Sarkar, A., Ghosh, S., and Konar, A. (2016). Robotic link position control using brain computer interface. In *2016 International Conference on Microelectronics, Computing and Communications (MicroCom)* (Durgapur, India: IEEE), 1–6. doi:10.1109/MicroCom.2016.7522567

Rotermund, D., Ernst, U. A., and Pawelzik, K. R. (2006). Towards On-line Adaptation of Neuro-prostheses with Neuronal Evaluation Signals. *Biological Cybernetics* 95, 243–257. doi:10.1007/s00422-006-0083-7

Salazar-Gomez, A. F., DelPreto, J., Gil, S., Guenther, F. H., and Rus, D. (2017). Correcting robot mistakes in real time using EEG signals. In *2017 IEEE International Conference on Robotics and Automation (ICRA)* (Singapore, Singapore: IEEE), 6570–6577. doi:10.1109/ICRA.2017.7989777

Schmidt, N. M., Blankertz, B., and Treder, M. S. (2012). Online detection of error-related potentials boosts the performance of mental typewriters. *BMC Neuroscience* 13, 19. doi:10.1186/1471-2202-13-19

Yousefi, R., Rezazadeh Sereshkeh, A., and Chau, T. (2019). Online detection of error-related potentials in multi-class cognitive task-based BCIs. *Brain-Computer Interfaces* 6, 1–12. doi:10.1080/2326263X.2019.1614770

Zhang, Y., Chen, W., Lin, C.-L., Chu, J., and Meng, F. (2018). Research on Command Confirmation Unit Based on Motor Imagery EEG Signal Decoding Feedback in Brain-Computer Interface. In *2018 15th International Conference on Control, Automation, Robotics and Vision (ICARCV)* (Singapore: IEEE), 1923–1928. doi:10.1109/ICARCV.2018.8581088
